# Unconditional stability of a recurrent neural circuit implementing divisive normalization

**Published:** 2025-01-15

**Authors:** Shivang Rawat, David J. Heeger, Stefano Martiniani

**Affiliations:** 1 Courant Institute of Mathematical Sciences, NYU; 2 Center for Soft Matter Research, Department of Physics, NYU; 3 Department of Psychology, NYU; 4 Center for Neural Science, NYU; 5 Simons Center for Computational Physical Chemistry, Department of Chemistry, NYU

## Abstract

Stability in recurrent neural models poses a significant challenge, particularly in developing biologically plausible neurodynamical models that can be seamlessly trained. Traditional cortical circuit models are notoriously difficult to train due to expansive nonlinearities in the dynamical system, leading to an optimization problem with nonlinear stability constraints that are difficult to impose. Conversely, recurrent neural networks (RNNs) excel in tasks involving sequential data but lack biological plausibility and interpretability. In this work, we address these challenges by linking dynamic divisive normalization (DN) to the stability of “oscillatory recurrent gated neural integrator circuits” (ORGaNICs), a biologically plausible recurrent cortical circuit model that dynamically achieves DN and that has been shown to simulate a wide range of neurophysiological phenomena. By using the indirect method of Lyapunov, we prove the remarkable property of unconditional local stability for an arbitrary-dimensional ORGaNICs circuit when the recurrent weight matrix is the identity. We thus connect ORGaNICs to a system of coupled damped harmonic oscillators, which enables us to derive the circuit’s energy function, providing a normative principle of what the circuit, and individual neurons, aim to accomplish. Further, for a generic recurrent weight matrix, we prove the stability of the 2D model and demonstrate empirically that stability holds in higher dimensions. Finally, we show that ORGaNICs can be trained by backpropagation through time without gradient clipping/scaling, thanks to its intrinsic stability property and adaptive time constants, which address the problems of exploding, vanishing, and oscillating gradients. By evaluating the model’s performance on RNN benchmarks, we find that ORGaNICs outperform alternative neurodynamical models on static image classification tasks and perform comparably to LSTMs on sequential tasks.

## 1 Introduction

Deep neural networks (DNNs) have found widespread use in modeling tasks from experimental systems neuroscience. The allure of DNN-based models lies in their ease of training and the flexibility they offer in architecting systems with desired properties [1–3]. In contrast, neurodynamical models like the Wilson-Cowan [4] or the Stabilized Supralinear Network (SSN) [5] are more biologically plausible than DNNs, but these models confront considerable training challenges due to the lack of stability guarantees for high-dimensional problems. Training recurrent neural networks (RNNs), by comparison, is more straightforward thanks to ad hoc regularization techniques like layer normalization, batch normalization, and gradient clipping/scaling, which help stabilize training without imposing strict stability constraints. Conversely, neurodynamical models require enforcing hard stability constraints while maintaining biological plausibility. In lower dimensions, it is relatively straightforward to derive constraints on model parameters that ensure a dynamically stable system [6, 7]. However, for high-dimensional systems, this becomes significantly more challenging, as integrating these hard constraints into the optimization problem is more complex [8, 9]. Stability is generally advantageous in DNNs, as it is linked to improved generalization, mitigation of exploding gradient problems, increased robustness to input noise, and simplified training techniques [10].

The divisive normalization (DN) model was developed to explain the responses of neurons in the primary visual cortex (V1) [11–14], and has since been applied to diverse cognitive processes and neural systems [15–24]. Therefore, DN has been proposed as a canonical neural computation [25] that is linked to many well-documented physiological [26, 27] and psychophysical [28, 29] phenomena. DN models various neural processes: adaptation [30, 31], attention [32], automatic gain control [33], decorrelation, and statistical whitening [34]. The defining characteristic of DN is that each neuron’s response is divided by a weighted sum of the activity of a pool of neurons (Eq. 2, below) like when normalizing the length of a vector. Due to its wide applicability and ability to explain a variety of neurophysiological phenomena, we argue that this characteristic should be central to any neurodynamical model. Both the Wilson-Cowan and SSN models have been shown to approximate DN responses [5, 35], but only approximately in certain parameter regimes.

Normalization techniques have been extensively adopted for training DNNs, demonstrating their ability to stabilize, accelerate training, and enhance generalization [36–38]. Divisive normalization can be viewed as a comprehensive normalization strategy, with batch and layer normalization being specific instances [39]. Models implementing DN have shown superior performance compared to common normalization methods (Batch [36], Layer [37], Group [40]) in tasks such as image recognition with convolutional neural networks (CNNs) [41] and language modeling with RNNs [39, 42]. Despite the foundational role of these techniques in deep learning algorithms, their implementation is ad hoc, limiting their conceptual relevance. They serve as practical solutions addressing the limitations of current machine learning frameworks rather than offering principled insights derived from understanding cortical circuits.

It has been proposed that DN is achieved via a recurrent circuit [11, 13, 43–47]. Oscillatory recurrent gated neural integrator circuits (ORGaNICs) are rate-based recurrent neural circuit models that implement DN dynamically via recurrent amplification [47, 48]. Since ORGaNICs’ response follows the DN equation at steady-state, its steady-state response captures the full range of aforementioned neural phenomena explained by DN [11–34]. ORGaNICs have further been shown to simulate key time-dependent neurophysiological and cognitive/perceptual phenomena under realistic biophysical constraints [47, 48]. Additional phenomena not explained by DN [49] can in principle be integrated into the model. In this paper, however, we focus on the effects of DN on the dynamical stability of ORGaNICs. Despite some empirical evidence that ORGaNICs are highly robust, the question of whether the model is stable for arbitrary parameter choices, and thus whether it can be robustly trained on ML tasks by backpropagation-through-time (BPTT), remains open.

Here, we establish the unconditional stability — applicable across all parameters and inputs — of a multidimensional two-neuron-types ORGaNICs model when the recurrent weight matrix is the identity. We prove this result, detailed in Section 4, by the indirect method of Lyapunov: we perform linear stability analysis around the model’s analytically-known normalization fixed point and reduce the stability problem to that of a high-dimensional mechanical system, whose stability is defined in terms of a tractable quadratic eigenvalue problem. We then address the stability of the model with an arbitrary recurrent weight matrix in Section 5. While the indirect method of Lyapunov becomes intractable for such a system, we provide proof of unconditional stability for a two-dimensional circuit with an arbitrary recurrent weight and offer empirical evidence supporting the claim of stability for high-dimensional systems.

ORGaNICs can be viewed as biophysically plausible extensions of Long Short Term Memory units (LSTMs) [3] and Gated Recurrent Units (GRUs) [50], RNN architectures that have been widely used in ML applications [3, 51–54]. The main differences are that ORGaNICs operate in continuous time and have built-in dynamic normalization (via recurrent gain modulation) and built-in attention (via input gain modulation). Thus, we expect that ORGaNICs should be able to solve relatively sophisticated tasks [47]. Here, we demonstrate (Section 6) that by virtue of their intrinsic stability, ORGaNICs can be trained on sequence modeling tasks by BPTT, in the same manner as traditional RNNs (unlike SSN that instead requires costly specialized training strategies [55]), despite implementing power-law activations [5]. Moreover, we show that ORGaNICs trained by naive BPTT (i.e., without gradient clipping/scaling or other ad hoc strategies) achieve performance comparable to LSTMs on the tasks that we consider, despite no systematic hyperparameter tuning.

## 2 Related Work

### Trainable biologically plausible neurodynamical models:

There have been several attempts to develop neurodynamical models that mimic the function of biological circuits and that can be trained on cognitive tasks. Song et al. [56] incorporated Dale’s law into the vanilla RNN architecture, which was successfully trained across a variety of cognitive tasks. Building on this, Soo et al. [57] developed a technique for such RNNs to learn long-term dependencies by using skip connections through time. ORGaNICs is a model that is already built on biological principles and can learn long-term dependencies intrinsically by tuning the (intrinsic or effective) time constants, therefore it does not require the method used in [57]. Soo et al. [55] introduced a novel training methodology (dynamics-neural growth) for SSNs and demonstrated its utility for tasks involving static (time-independent) stimuli. However, this training approach is costly and difficult to scale (because SSNs, unlike ORGaNICs, are not unconditionally stable), and its applicability on tasks with dynamically changing inputs remains unclear.

### Dynamical systems view of RNNs:

The stability of continuous-time RNNs has been extensively studied and discussed in a comprehensive review by Zhang et al. [58]. Recent advancements have focused on designing architectures that address the issues of vanishing and exploding gradients, thereby enhancing trainability and performance. A central idea in these designs is to achieve better trainability and generalization by ensuring the dynamical stability of the network. Moreover to avoid the problem of vanishing gradients the key idea is to constrain the real part of the eigenvalues of the linearized dynamical system to be close to zero, which facilitates the propagation and retention of information over long durations of time. Chang et al. [59] and Erichson et al. [60] achieve this by imposing an antisymmetric constraint on the recurrent weight matrix. Meanwhile, Rusch et al. [61, 62] propose an architecture based on coupled damped harmonic oscillators, resulting in a second-order system of ordinary differential equations that behaves similarly to how ORGaNICs behave in the vicinity of the normalization fixed point, as we show in Section 4. Despite their impressive performance on various sequential data benchmarks, these models lack biological plausibility due to their use of saturating nonlinearities (instead of normalization) and unrealistic weight parameterizations.

## 3 Model description

In its simplest form, the two-neuron-types ORGaNICs model [47, 48] with n neurons of each type can be written as,

(1) $$\tau_{y}⊙\dot{\text{y}}=-y+b⊙z+\left(1-a^{+}\right)⊙\left(W_{r}\left(\sqrt{y^{+}}-\sqrt{y^{-}}\right)\right)\;\tau_{a}⊙\dot{a}=-a+b_{0}^{2}⊙\sigma^{2}+W\left(\left(y^{+}+y^{-}\right)⊙a^{+2}\right)$$

where $y\in R^{n}$ and $a\in R^{n}$ are the membrane potentials (relative to an arbitrary threshold potential that we take to be 0) of the excitatory $\left(y\right)$ and inhibitory $\left(a\right)$ neurons, evolving according to the dynamical equations defined above with $\dot{y}$ and $\dot{a}$ denoting the time derivatives. The notation ⊙ denotes element-wise multiplication of vectors, and squaring, rectification, square-root, and division are also performed element-wise. 1 is an n-dimensional vector with all entries equal to 1. $z\in R^{n}$ is the input drive to the circuit and is a weighted sum of the input, $x\in R^{m}$, i.e., $z=W_{zx}x$. The firing rates, $y^{\pm }=\lfloor \pm y\rfloor^{2}$ and $a^{+}=\sqrt{\left\lfloor a\right\rfloor }$ are rectified $(\left.\left\lfloor .\right\rfloor \right)$ power functions of the underlying membrane potentials. For the derivation of a general model with arbitrary power-law exponents, including the Eq. 1, see Appendix A. Note that the term $\sqrt{y^{+}}-\sqrt{y^{-}}$ serves the purpose of defining a mechanism for reconstructing the membrane potential (which can be negative, depending on the sign of the input) from the firing rates $y^{\pm }$ that are strictly nonnegative. $y^{+}$ and $y^{-}$ are the firing rates of neurons with complementary receptive fields such that they encode inputs with positive and negative signs, respectively. Note that only one of these neurons fires at a given time. In ORGaNICs, these neurons have a single dynamical equation for their membrane potentials, where the sign of y indicates which neuron is active. Neurons with such complementary (anti-phase) receptive fields are found adjacent to each other in the visual cortex [63], and we hypothesize that such complementary neurons are ubiquitous throughout the neocortex. $b\in R_{*}^{+n}$ and $b_{0}\in R_{*}^{+n}$ are the input gains for the external inputs z and σ fed to neurons y and a, respectively. $R_{*}^{+}$ is the set of positive real numbers, $\left\{x\in R|x>0\right\}.$ $\sigma \in R_{*}^{+n}$ determines the semisaturation of the responses of neurons y by contributing to the depolarization of neurons a. $\tau_{y}\in R_{*}^{+n}$ and $\tau_{a}\in R_{*}^{+n}$ represent the time constants of y and a neurons.

In addition to receiving external inputs, both y and a neurons receive recurrent inputs, represented by the last term in both of the equations. $W_{r}\in R^{n\times n}$ is the recurrent weight matrix that captures lateral connections between the y neurons. This recurrent input is gated by the a neurons, via the term $\left(1-a^{+}\right)$. Similarly, the *nonnegative* normalization weight matrix, $W\in {R_{*}}^{n\times n}$, encapsulates the recurrent inputs received by the a neurons. The differential equations are designed in such a way that when $W_{r}=I$ and $b=b_{0}=b_{0}1$ (i.e., with all elements equal to a constant $b_{0}$), the principal neurons follow the normalization equation exactly (and approximately when $W_{r}\neq I$) at steady-state,

(2) $$y_{s}^{+}\equiv {\left\lfloor y_{s}\right\rfloor }^{2}=\frac{\lfloor z\rfloor^{2}}{\sigma^{2}+W\left(\lfloor z\rfloor^{2}+\lfloor -z\rfloor^{2}\right)}.$$

$\lfloor z\rfloor^{2}$ and $\lfloor -z\rfloor^{2}$ represent the contribution of neurons with complementary receptive fields to the normalization pool, and $\lfloor z\rfloor^{2}+\lfloor -z\rfloor^{2}=z^{2}$ is the contrast energy of the input. Note that the recurrent gain, $\left(1-a^{+}\right)$, is a particular nonlinear function of the output responses/activation designed to achieve DN, while the input gain, $b^{+}$, is an input gate that can implement an attention mechanism.

## 4 Stability analysis of high-dimensional model with identity recurrent weights

We consider the stability of the general high-dimensional ORGaNICs (Eq. 1) when the recurrent weight matrix is identity, $W_{r}=I$. We first simplify the dynamical system by noting that $\sqrt{y^{+}}-\sqrt{y^{-}}=y$ and $y^{+}+y^{-}=y^{2}$ yielding the following equations,

(3) $$\tau_{y}⊙\dot{\text{y}}=-\sqrt{\left\lfloor a\right\rfloor }⊙y+b⊙z\;\tau_{a}⊙\dot{a}=-a+b_{0}^{2}⊙\sigma^{2}+W\left(y^{2}⊙\left\lfloor a\right\rfloor \right)$$

For identity recurrent weights, we have a unique fixed point, given by,

(4) $$y_{s}=\frac{b⊙z}{\sqrt{b_{0}^{2}⊙\sigma^{2}+W\left(b^{2}⊙z^{2}\right)}};\;a_{s}=b_{0}^{2}⊙\sigma^{2}+W\left(b^{2}⊙z^{2}\right)$$

Since the normalization weights in the matrix W are nonnegative, at steady-state we have $a_{s}>0$, so that $\sqrt{\left\lfloor a_{s}\right\rfloor }=\sqrt{a_{s}}$, and the corresponding firing rates at steady-state are,

(5) $$y_{s}^{\pm }=\frac{\lfloor \pm b⊙z\rfloor^{2}}{b_{0}^{2}⊙\sigma^{2}+W\left(b^{2}⊙z^{2}\right)};\;a_{s}^{+}=\sqrt{b_{0}^{2}⊙\sigma^{2}+W\left(b^{2}⊙z^{2}\right)}$$

Note that we recover the normalization equation, Eq. 2, if $b=b_{0}=b_{0}1$. Since the fixed points of y and a neurons are known analytically, to prove that this fixed point is *locally asymptotically stable* (i.e., the responses converge asymptotically to the fixed point), we apply the *indirect method of Lyapunov* at this fixed point [64]. This method allows us to analyze the stability of the nonlinear system in the vicinity of the fixed point by studying the corresponding linearized system. The Jacobian matrix $J\in R^{2n\times 2n}$ about $\left(y_{s},a_{s}\right)$, defining the linearized system, is given by,

(6) $$J=\left[\begin{array}{cc} -D\left(\frac{\sqrt{a_{s}}}{\tau_{y}}\right) & -D\left(\frac{y_{s}}{2⊙\sqrt{a_{s}}⊙\tau_{y}}\right)\\ D\left(\frac{2}{\tau_{a}}\right)WD\left(a_{s}⊙y_{s}\right) & D\left(\frac{1}{\tau_{a}}\right)\left(-I+WD\left(y_{s}^{2}\right)\right) \end{array}\right]$$

where D(x) is a diagonal matrix of appropriate size with the elements of the vector x on the diagonal. A necessary and sufficient condition for local stability is that the real parts of all eigenvalues of this matrix are negative. We thus proceed by computing the characteristic polynomial for the Jacobian, $p_{J}(\lambda )\equiv \text{det}(J-\lambda I)$. The roots of this polynomial, found by setting $p_{J}(\lambda )=0$, are the eigenvalues of the system. Consider the block matrix,

(7) $$J-\lambda I=\left[\begin{array}{ll} A_{11} & A_{12}\\ A_{21} & A_{22} \end{array}\right]=\left[\begin{array}{cc} -D\left(\frac{\sqrt{a_{s}}}{\tau_{y}}\right)-\lambda I & -D\left(\frac{y_{s}}{2⊙\sqrt{a_{s}}⊙\tau_{y}}\right)\\ D\left(\frac{2}{\tau_{a}}\right)WD\left(a_{s}⊙y_{s}\right) & D\left(\frac{1}{\tau_{a}}\right)\left(-I+WD\left(y_{s}^{2}\right)\right)-\lambda I \end{array}\right]$$

Notice that $A_{11}$ and $A_{12}$ are diagonal and therefore they commute, i.e., $A_{11}A_{12}=A_{12}A_{11}$, so we have that $\text{det}(J-\lambda I)=\text{det}\left(A_{22}A_{11}-A_{21}A_{12}\right)$ which is a property of the determinant of block matrices with commuting blocks [65]. Therefore, the characteristic polynomial of the linearized system after expansion of the terms and simplification is given by,

(8) $$\text{det}(J-\lambda I)=\text{det}\left(\lambda^{2}I+\lambda \left[D\left(\frac{1}{\tau_{a}}\right)+D\left(\frac{\sqrt{a_{s}}}{\tau_{y}}\right)-D\left(\frac{1}{\tau_{a}}\right)WD\left(y_{s}^{2}\right)\right]+D\left(\frac{\sqrt{a_{s}}}{\tau_{y}⊙\tau_{a}}\right)\right)$$

Finding the roots of this polynomial is thus a quadratic eigenvalue problem of the form $ℒ(\lambda )\equiv \text{det}\left(\lambda^{2}I+\lambda B+K\right)=0$, which has been studied extensively [66–69]. ℒ(λ) can be interpreted as the characteristic polynomial associated with a system of linear second-order differential equations with constant coefficients of the form $I\ddot{x}+B\dot{x}+Kx=0$. Therefore, proving the stability of our system (i.e., Re(λ)<0 for {λ:ℒ(λ)=0}), is equivalent to proving the asymptotic stability of $I\ddot{x}+B\dot{x}+Kx=0$.

Tisseur et al. [67] and Kirillov et al. [69] list a set of constraints on the damping matrix, B, and stiffness matrix, K, that yield a stable system, but they are *not* directly applicable to our system. In the context of a high-dimensional mechanical system, our system falls under the category of *gyroscopically stabilized systems with indefinite damping*. Few results are known about the conditions leading to the stability of such systems. By constructing a Lyapunov function, we prove (Appendix B) the following stability theorem that is directly applicable to our system, following an approach similar to Kliem et al. [70].

**Theorem 4.1.** *For a system of linear differential equations with constant coefficients of the form*,

(9) $$I\ddot{x}+B\dot{x}+Kx=0$$

*where* $B\in R^{n\times n}$ *and* $K\in R^{n\times n}$ *is a positive diagonal matrix (hence K≻0*), *the dynamical system is globally asymptotically stable if B is Lyapunov diagonally stable*.

Since the stiffness matrix,

(10) $$K=D\left(\frac{\sqrt{a_{s}}}{\tau_{y}⊙\tau_{a}}\right)=D\left(\frac{\sqrt{b_{0}^{2}⊙\sigma^{2}+W\left(b^{2}⊙z^{2}\right)}}{\tau_{y}⊙\tau_{a}}\right)$$

is a positive diagonal matrix, a sufficient condition for stability of the system is that the damping matrix, B, given by,

(11) $$B=B_{1}+B_{2}-B_{3}\;=D\left(\frac{1}{\tau_{a}}\right)+D\left(\frac{\sqrt{a_{s}}}{\tau_{y}}\right)-D\left(\frac{1}{\tau_{a}}\right)WD\left(y_{s}^{2}\right)\;=D\left(\frac{1}{\tau_{a}}\right)+D\left(\frac{\sqrt{b_{0}^{2}⊙\sigma^{2}+W\left(b^{2}⊙z^{2}\right)}}{\tau_{y}}\right)-D\left(\frac{1}{\tau_{a}}\right)WD\left(\frac{b^{2}⊙z^{2}}{b_{0}^{2}⊙\sigma^{2}+W\left(b^{2}⊙z^{2}\right)}\right)$$

is *Lyapunov diagonally stable*, i.e., there exists a positive definite diagonal matrix T, such that $TB+B^{⊤}T$ is positive definite.

Since all of the parameters are positive, and the weights in the matrix W are nonnegative, we can conclude the following: $B_{1}$ and $B_{2}$ are positive diagonal matrices and $B_{3}$ is a matrix with all positive entries (that may or may not be symmetric). Therefore, B is a *Z-matrix*, meaning that its off-diagonal entries are nonpositive. Further, a *Z-matrix* is *Lyapunov diagonally stable* if and only if it is a nonsingular *M-matrix*. Intuitively, *M-matrices* are matrices with non-positive off-diagonal elements and “large enough” positive diagonal entries. Berman & Plemmons [71] list 50 equivalent definitions of nonsingular *M-matrices*. We use the one that is best suited for our problem,

**Theorem 4.2.** *(Chapter 6, Theorem 2.3 from* [71]*) A Z-matrix matrix* $B\in R^{n\times n}$ *is Lyapunov diagonally stable if and only if there exists a convergent regular splitting of the matrix, that is, it has a representation of the form B=M-N*, *where* $M^{-1}$ *and* N *have all nonnegative entries, and $M^{-1}N$ has a spectral radius smaller than 1*.

We now show that, indeed, B has a *convergent regular splitting* for all combinations of the circuit parameters and for all inputs. We have already shown that B is a *Z-matrix*, therefore, the first condition of the theorem is satisfied. Next, we consider the following splitting B=M-N with $M=B_{1}+B_{2}$ and $N=B_{3}$. Since $B_{1}$ and $B_{2}$ are positive diagonal matrices, $M^{-1}$ is nonnegative, while N is also nonnegative because $B_{3}$ has all positive entries. Therefore, the only condition left to satisfy is that the spectral radius of $M^{-1}N$ is smaller than 1, or that the matrix is convergent.

The matrix $S=M^{-1}N={\left(B_{1}+B_{2}\right)}^{-1}B_{3}$ can be written as,

(12) $$S=D\left(\frac{1}{1+\left(\tau_{a}/\tau_{y}\right)⊙\sqrt{b_{0}^{2}⊙\sigma^{2}+W\left(b^{2}⊙z^{2}\right)}}\right)WD\left(\frac{b^{2}⊙z^{2}}{b_{0}^{2}⊙\sigma^{2}+W\left(b^{2}⊙z^{2}\right)}\right)$$

We prove the following theorem (Appendix D) which directly applies to S,

**Theorem 4.3.** *A matrix* A *of the form $A=D\left(t\right)WD(u/(v+Wu))$ is convergent (i.e., its spectral radius is less than 1), if $W\in R^{n\times n}$ and* $t,u,v\in R^{n}$ *satisfy* $0<t_{i}<1,u_{i}\geq 0,v_{i}>0$ *and* $w_{ij}\geq 0$ *for all i, j*.

Defining $t\to 1/\left(1+\left(\tau_{a}/\tau_{y}\right)⊙\sqrt{b_{0}^{2}⊙\sigma^{2}+W\left(b^{2}⊙z^{2}\right)}\right),u\to b^{2}⊙z^{2}$ and $v\to b_{0}^{2}⊙\sigma^{2}$, it can be seen that they satisfy the constraints of the theorem, and thus S is convergent. This implies that B has a *convergent regular splitting* and, as a result, the linearized dynamical system is unconditionally globally asymptotically stable for all the values of parameters and inputs. Further, the global asymptotic stability of linearization implies the local asymptotic stability of the normalization fixed point for ORGaNICs.

This result holds even when the neurons have different time constants, regardless of their type, as no assumptions were made about the time constants. This finding is significant for machine learning, particularly for designing architectures based on ORGaNICs. It allows neurons/units to integrate information at varying time scales while maintaining a stable circuit that performs normalization dynamically. Moreover, analytical expressions for eigenvalues can be obtained in the following case,

**Theorem 4.4.** *Let* $W_{r}=I$, *the normalization matrix be given by* W=αE, *where* E *is the all-ones matrix, and the parameters are scalars, i.e*., $\tau_{y}=\tau_{y}1,\tau_{a}=\tau_{a}1,b_{0}=b_{0}1$, *and* σ=σ1. *Under these conditions, the eigenvalues of the system admit closed form solutions (detailed in* Appendix C).

This result is particularly useful for neuroscience as it elucidates the connection between ORGaNICs parameters and the strength and frequency of oscillatory activity. Since we followed a direct Lyapunov approach to prove Theorem 4.1 as shown in Appendix B, we can derive an *energy* (viz., Lyapunov function) for ORGaNICs as shown in Appendix H.

**Theorem 4.5.** *When* $W_{r}=I$, *the energy (Lyapunov function) minimized by ORGaNICs in the vicinity of the normalization fixed point, is given by*,

(13) $$V(y,a)=\sum_{i=1}^{n}t_{i}\frac{a_{si}}{{y_{si}}^{2}}\left[\frac{\tau_{y_{i}}}{\tau_{ai}}\sqrt{a_{si}}{\left(y_{i}-y_{si}\right)}^{2}+{\left(\sqrt{a_{i}}y_{i}-\sqrt{a_{si}}y_{si}\right)}^{2}\right].$$

*Where* $t_{i}$ *are the diagonal entries of T and* $y_{s_{i}}\left(a_{si}\right)$ *are the steady-state values of neurons $y_{i}\left(a_{i}\right)$*.

Specifically, for a two-dimensional model (one y neuron and one a neuron) this expression simplifies to reveal that ORGaNICs behave like a damped harmonic oscillator with *energy*,

(14) $$V(y,a)=\frac{\tau_{y}}{\tau_{a}}\sqrt{b_{0}^{2}\sigma^{2}+wb^{2}z^{2}}{\left(y-\frac{bz}{\sqrt{b_{0}^{2}\sigma^{2}+wb^{2}z^{2}}}\right)}^{2}+(\sqrt{a}y-bz{)}^{2}$$

This result demonstrates that ORGaNICs minimize the residual of the instantaneously reconstructed gated input drive $\left(\sqrt{a}y-bz\right)$, while also ensuring that the principal neuron’s response, y, achieves DN. The balance between these objectives is governed by the parameters and the external input strength. With fixed parameters, weaker inputs, z, cause the model to prioritize input matching over normalization, whereas stronger inputs increasingly engage the normalization objective.

## 5 Stability analysis for arbitrary recurrent weights

Now, we relax the constraint that the recurrent weight matrix must be identity, allowing $W_{r}\neq I$, and see how the stability result changes. This leads to the following set of equations,

(15) $$\tau_{y}⊙\dot{\text{y}}=-y+b⊙z+\left(1-\sqrt{\left\lfloor a\right\rfloor }\right)⊙\left(W_{r}y\right)\;\tau_{a}⊙\dot{a}=-a+b_{0}^{2}⊙\sigma^{2}+W\left(y^{2}⊙\left\lfloor a\right\rfloor \right)$$

The linear stability analysis becomes intractable for a general $W_{r}$ because we no longer have a closed-form analytical expression for the steady states of y and a. Additionally, the characteristic polynomial cannot be expressed in a way similar to Eq.8. Nevertheless, for a two-dimensional system,

(16) $$\tau_{y}\dot{y}=-y+bz+\left(1-\sqrt{\left\lfloor a\right\rfloor }\right)w_{r}y\;\tau_{a}\dot{a}=-a+b_{0}^{2}\sigma^{2}+wy^{2}\left\lfloor a\right\rfloor$$

we can prove the following, with a detailed analysis provided in Appendix E.

**Theorem 5.1.** *Given that the recurrence is contracting, i.e.,* $0<w_{r}\leq 1$, *when* z>0(z<0) *there exists a unique fixed point with $y_{s}>0\left(y_{s}<0\right)$ and* $a_{s}>0$, *and it is asymptotically stable*.

**Theorem 5.2.** *Given that the recurrence is expansive, i.e.,* $w_{r}>1$, *there are either 1 or 3 fixed points of which at least one is asymptotically stable. When* z>0(z<0) *there exists exactly 1 fixed point with $y_{s}>0\left(y_{s}<0\right)$ and* $a_{s}>0$, *and it is asymptotically stable. If $b_{0}\sigma >1-1/w_{r}$*, *there are no additional fixed points. If $b_{0}\sigma <1-1/w_{r}$*, *there exist either* 0 *or* 2 *additional fixed points with $y_{s}<0\left(y_{s}>0\right)$ and* $a_{s}>0$ *whose stability cannot be guaranteed*.

**Figure 1:**
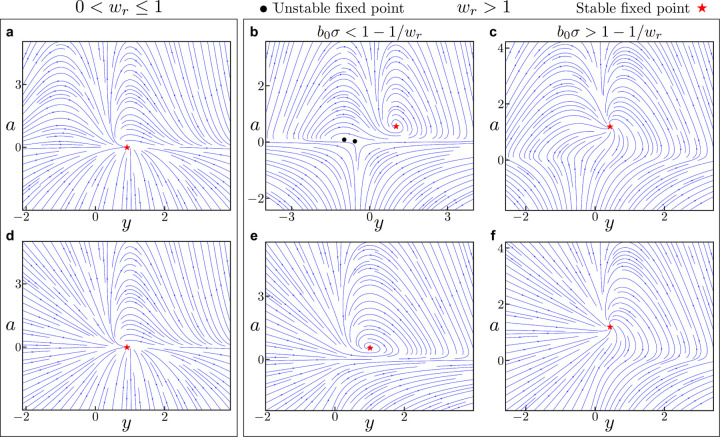
Phase portraits for 2D ORGaNICs with positive input drive. We plot the phase portraits of 2D ORGaNICs in the vicinity of the stable fixed points for contractive (**a**, **d**) and expansive (**b, c, e, f**) recurrence scalar $w_{r}$. A stable fixed point always exists, regardless of the parameter values. (**a-c**), The main model (Eq. 16). (**d-f**), The rectified model (Eq. 102). Red stars and black circles indicate stable and unstable fixed points, respectively. The parameters for all plots are: $b=0.5,\tau_{a}=2\;\text{ms},\tau_{y}=2\;\text{ms},w=1.0$, and z=1.0. For **(a) & (d)**, the parameters are $w_{r}=0.5,b_{0}=0.5,\sigma =0.1$; for **(b) & (e)**, $w_{r}=2.0,b_{0}=0.5,\sigma =0.1$; and for **(c) & (f)**, $w_{r}=2.0,b_{0}=1.0,\sigma =1.0$.

We plot the phase portraits for these different cases in Fig. 1. The key takeaway is that there is always a fixed point $\left(y_{s},a_{s}\right)$ with $a_{s}>0$ and $y_{s}$ having the same sign as z. This fixed point is asymptotically stable regardless of the value of $w_{r}$. Based on these results and the proven stability of arbitrary dimensional ORGaNICs when $W_{r}=I$ (as shown in Section 4), we conjecture that

**Conjecture 5.3.** *Consider high-dimensional ORGaNICs with an arbitrary recurrent weight matrix* $W_{r}$ *and no constraints on the remaining parameters. If the norm of the input drive satisfies ‖z‖≤1*, *and the maximum singular value of $W_{r}$ is constrained to be 1, then the system possesses at least one asymptotically stable fixed point*.

This conjecture is supported by empirical evidence showing consistent stability, as ORGaNICs initialized with random parameters and inputs under these constraints have exhibited stability in 100% of trials, see Fig. 4. We further speculate that ORGaNICs may be *typically* stable beyond this regime as we find that 100% of trials yield a stable circuit when the constraint on the maximum singular value of $W_{r}$ is increased to 2, but it becomes unstable when it is increased to 3.


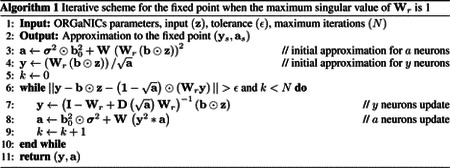


## 6 Experiments

We provide further empirical evidence in support of Conjecture 5.3 that ORGaNICs is asymptotically stable by showing that stability is preserved when training ORGaNICs using naïve BPTT on two different tasks: 1) static classification of MNIST, 2) sequential classification of pixel-by-pixel MNIST. Because these ML tasks have no relevance for neurobiological or cognitive processes, we relax one aspect of the biological plausibility of ORGaNICs, specifically, allowing arbitrary (learned) nonnegative values for the intrinsic time constants.^1^

### 6.1 Static input classification task

We first show that we can train ORGaNICs on the MNIST handwritten digit dataset [72] presented to the circuit as a static input. This setting corresponds to evolving the responses of the neurons dynamically until they reach a fixed point solution and using the steady-state firing rates of the principal neurons to predict the labels, akin to deep equilibrium models [73]. While the fixed point of the circuit is known when $W_{r}=I$ (given by Eq. 89), we allow $W_{r}$ to be learnable and parameterized it to have a maximum singular value of 1. This constraint allows us to find the fixed point responses of all the neurons without simulation, using a fixed point iteration scheme (Algorithm 1) that converges with great accuracy in a few (less than 5) steps, see Fig. 4 & 5. We provide an intuition for why this algorithm works with empirical evidence of fast convergence in Appendix G.

We trained ORGaNICs on this task (details provided in Appendix I.1) and compared its performance to SSN [5] trained by dynamics-neutral growth [55]. We found that ORGaNICs perform better than SSN with the same model size, and on par with an MLP (Table 1). We analyzed the eigenvalues of the Jacobian matrix of the trained model and consistently found the largest real part to be negative (Fig. 5), indicating stability. Moreover, we found that stability was maintained during training (Fig. 6).

### 6.2 Time varying input

We trained unconstrained ORGaNICs by naïve BPTT on a classification task of sequential MNIST (sMNIST), proposed by Le et al. [74]. This is a challenging task because it involves long-term dependencies and requires the architecture to maintain and integrate information over long timescales. Briefly, the task involves the presentation of pixels of MNIST images sequentially (one pixel for each timestep) in scanline order, and at the end of the input the model has to predict the digit that was presented. There is a more complicated version of this task, permuted sequential MNIST, in which the pixels of all images are permuted in some random order before being presented sequentially. We train ORGaNICs with different hidden layer sizes (number of y neurons) on these two tasks by discretizing the rectified ORGaNICs with arbitrary recurrence, Eq. 87, which has all the properties that we have derived for the main model. Since an unstable fixed point is undesirable in such a task, as it may lead to diverging trajectories, we prefer the rectified model (Appendix F) over the main model. We proved that the 2D rectified ORGaNICs (Eq. 102) does not exhibit an unstable fixed point for positive inputs, as it can also be seen in Fig 1. The hidden states of the neurons are initialized with a uniform random distribution (for more details, see Appendix I.2). Additionally, we make the input gains b and $b_{0}$ dynamical with their ODEs given by,

(17) $$\tau_{b}⊙\dot{b}=-b+f\left(W_{bx}x+W_{by}y+W_{ba}a\right)\;\tau_{b_{0}}⊙{\dot{b}}_{0}=-b_{0}+f\left(W_{b_{0}x}x+W_{b_{0}y}y+W_{b_{0}a}a\right)$$

We achieved slightly better performance than LSTMs on sMNIST with a smaller model size and comparable performance on permuted sMNIST, without hyperparameter optimization and without gradient clipping/scaling (Table 2). We found that the trajectories of y are bounded when it is trained on the sequential task (Fig. 7), indicating stability. We also show that the training of ORGaNICs is stable and does not require gradient clipping when the intrinsic time constants of the neurons are fixed (Table 2).

## 7 Discussion

### Summary:

While extensive research has been aimed at identifying highly expressive RNN architectures that can model complex data, there has been little advancement in developing robust, biologically plausible recurrent neural circuits that are easy to train and perform comparably to their artificial counterparts. Regularization techniques such as batch, group, and layer normalization have been developed and are implemented as ad hoc add-ons making them biologically implausible. In this work, we bridge these gaps by leveraging the recently proposed ORGaNICs model which implements divisive normalization (DN) dynamically in a recurrent circuit. We establish the unconditional stability of an arbitrary dimensional ORGaNICs circuit with an identity recurrent weight matrix $\left(W_{r}\right)$, with all of the other parameters and inputs unconstrained, and provide empirical evidence of stability for ORGaNICs with arbitrary $W_{r}$. Since ORGaNICs remain stable for all parameter values and inputs, we do not need to resort to techniques that are restrictive in parameter space, or that require designing unrealistic structures for weight matrices. ORGaNICs’ intrinsic stability mitigates the issues of exploding and oscillating gradients, enabling the use of “vanilla” BPTT without the need for gradient clipping, which is instead required when training LSTMs. Moreover, ORGaNICs effectively address the vanishing gradient problem often encountered when training RNNs. This is achieved by processing information across various timescales, resulting in a blend of lossy and non-lossy neurons, while preserving stability. The model’s effectiveness in overcoming vanishing gradients is further evidenced by its competitive performance against architectures specifically designed to address this issue, such as LSTMs.

### Dynamic normalization:

Normalization techniques, such as batch and layer normalization, are fundamental in modern ML architectures significantly enhancing the training and performance of CNNs. However, a principled approach to incorporating normalization into RNNs has remained elusive. While layer normalization is commonly applied to RNNs to stabilize training, it does not influence the underlying circuit dynamics since it is applied a-posteriori to the output activations, leaving the stability of RNNs unaffected. Furthermore, DN has been shown to generalize batch and layer normalization [39], leading to improved performance [39, 41, 42]. ORGaNICs, unlike RNNs with layer normalization, implement DN dynamically within the circuit, marking the first instance of this concept being applied and analyzed in ML. Our work demonstrates that embedding DN within a circuit naturally leads to stability, which is greatly advantageous for trainability. This stability, a consequence of dynamic DN, sets ORGaNICs apart from other RNNs by providing both output normalization and model robustness. As a result, ORGaNICs can be trained using BPTT, achieving performance on par with LSTMs. The key insight is that the dynamic application of DN not only enhances training efficiency but also improves model robustness. This illustrates how the incorporation of neurobiological principles can drive advances in ML.

### Interpretability:

In the proof of stability, we establish a direct connection between ORGaNICs and systems of coupled damped harmonic oscillators, which have long been studied in mechanics and control theory. This analogy not only enables us to derive an interpretable energy function for ORGaNICs (Eq. 13), providing a normative principle of what the circuit aims to accomplish, but also sheds light on the link between normalization and dynamical stability of neural circuits. For a relevant ML task, having an analytical expression for the energy function allows us to quantify the relative contributions of the individual neurons in the trained model, offering more interpretability than other RNN architectures. For instance, Eq. 13 shows that the ratio of time constants $\left(\tau_{y}/\tau_{a}\right)$ for E-I neuron pairs determines how much weight a neuron assigns to divisive normalization relative to aligning its responses with the input drive z. This insight provides a clear functional role for each neuron in the trained model. Moreover, since ORGaNICs are biologically plausible, we can understand how the various components of the dynamical system might be computed within a neural circuit [48], bridging the gap between theoretical models and biological implementation, and offering a means to generate and test hypotheses about neural computation in real biological systems (which we will be reporting elsewhere).

### Limitations:

Although the stability property pertains to a continuous-time system of nonlinear differential equations, typical implementations for tasks with sequential data involve an Euler discretization of these equations for training purposes. This might lead to a stiff dynamical system, potentially causing numerical instabilities and explosive dynamics, highlighting the importance of carefully parameterizing time constants and choosing a small enough time step to maintain stable dynamics. The proof of unconditional stability is only tractable for the two-dimensional circuit and the high-dimensional circuit with $W_{r}=I$. Therefore, we can only conjecture the stability of ORGaNICs for arbitrary $W_{r}$, based on these two limiting cases and on empirical evidence. In the current form, the weight matrices of the input gain modulators, $W_{by},W_{ba},W_{b_{0}y}$, and $W_{b_{0}a}$, are each n×n. As a result, the number of parameters grows more rapidly with the hidden state size compared to other RNNs. To mitigate this, we plan to explore using compact and/or convolutional weights to prevent a significant increase in the number of parameters as the hidden state size expands.

### Attention mechanisms in ORGaNICs:

ORGaNICs have a built-in mechanism for attention: modulating the input gain b (e.g., Eq. 17), coupled with DN. This attention mechanism aligns with experimental data on both increases in the gain of neural responses and improvements in behavioral performance [19,20,32,76–85]. Moreover, this mechanism performs a computation that is analogous to that of an attention head in ML systems (including transformers [2]) as both operate by changing the gain over time. In ORGaNICs, DN replaces the softmax operation typically used in an attention head.

### Future work:

This study has explored only a single layer of ORGaNICs for the sequential tasks. Future work will examine how stacked layers with feedback connections, similar to those in the cortex, perform on benchmarks for sequential modeling and also on cognitive tasks with long-term dependencies. We have thus far shown that ORGaNICs can address the problem of long-term dependencies by learning intrinsic time constants. Future investigations will assess the performance of ORGaNICs for tasks with long-term dependencies by learning to modulate the responses of the a and b neurons to control the effective time constant of the recurrent circuit (without changing the intrinsic time constants) [47], i.e., implementing a working memory circuit capable of learning to maintain and manipulate information across various timescales.

## Supplementary Material

1

## Figures and Tables

**Table 1:** Test accuracy on MNIST dataset

Model	Accuracy
SSN (50:50)	94.9%
SSN (80:20)	95.2%
MLP (50)	98.2%
**ORGaNICs** (50:50)	98.1%
**ORGaNICs** (80:80)	98.2%
**ORGaNICs** (two layers)	98.1%

**Table 2:** Test accuracy on sequential pixel-by-pixel MNIST and permuted MNIST

Model	sMNIST	psMNIST	# units	# params
LSTMs [75]	97.3%	92.6%	128	68k
AntisymmetricRNN [59]	98.0%	95.8%	128	10k
coRNN [61]	99.3%	96.6%	128	34k
Lipschitz RNN [60]	99.4%	96.3%	128	34k
**ORGaNICs** (fixed time constants)	90.3%	80.3%	64	26k
**ORGaNICs** (fixed time constants)	94.8%	84.8%	128	100k
**ORGaNICs**	97.7%	89.9%	64	26k
**ORGaNICs**	97.8%	90.7%	128	100k

## Footnotes

1 Python code for this study is available at https://github.com/martiniani-lab/dynamic-divisive-norm.
