# Supplementary material for: Unconditional stability of a recurrent neural circuit implementing divisive normalization
Source: ArXiv. 2024 Oct 31:arXiv:2409.18946v2. Originally published 2024 Sep 27. Preprint. [Version 2] (PMC11469413)
Supplement: Supplementary file 1 [file NIHPP2409.18946v2-supplement-1.pdf]

J Supplementary figures

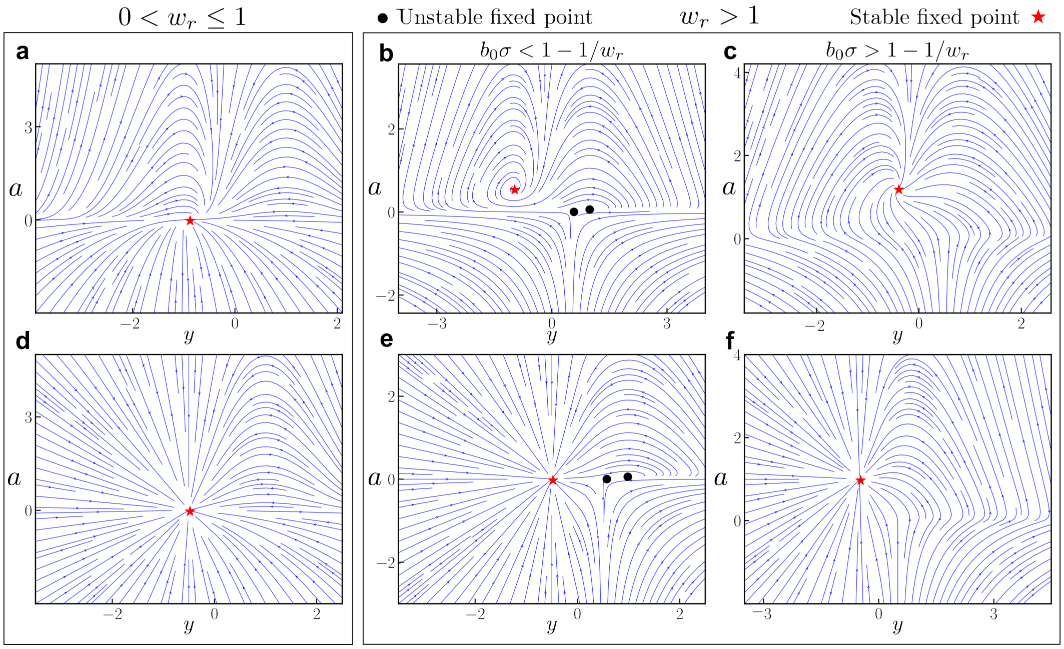

Figure 2: **Phase portraits for 2D ORGaNICs with negative input drive.** We plot the phase portraits of 2D ORGaNICs in the vicinity of the stable fixed point for contractive (**a, d**) and expansive (**b, c, e, f**) recurrence scalar w_r. A stable fixed point always exists, regardless of the parameter values. **(a-c)**, The main model (Eq. 16). **(d-f)**, The rectified model (Eq. 102). Red stars and black circles indicate stable and unstable fixed points, respectively. The parameters for all plots are: $b = 0.5$, $\tau_a = 2\,\text{ms}$, $\tau_y = 2\,\text{ms}$, $w = 1.0$, and $z = -1.0$. For **(a) & (d)**, the parameters are $w_r = 0.5$, $b_0 = 0.5$, $\sigma = 0.1$; for **(b) & (e)**, $w_r = 2.0$, $b_0 = 0.5$, $\sigma = 0.1$; and for **(c) & (f)**, $w_r = 2.0$, $b_0 = 1.0$, $\sigma = 1.0$.

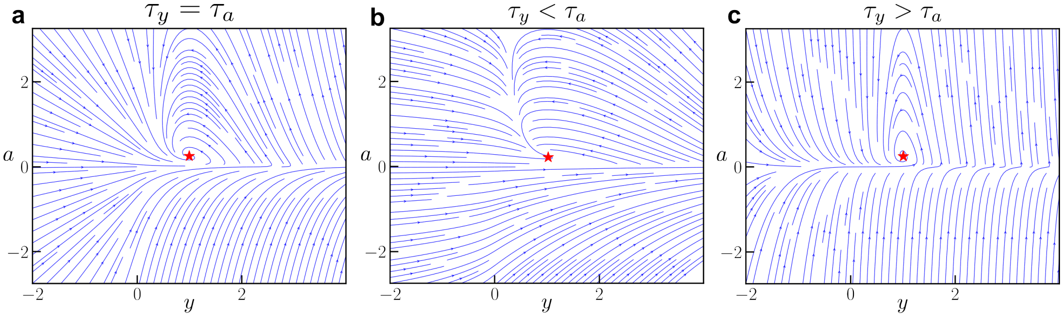

Figure 3: **Phase portraits for 2D rectified ORGaNICs for different time constants.** Red stars indicate stable fixed points. The parameters for all plots are: $w_r = 1.0$, $b_0 = 0.5$, $b = 0.5$, $\sigma = 0.1$, $w = 1.0$, and $z = 1.0$. For **(a)**, the time constants are $\tau_a = 2\,\text{ms}$, $\tau_y = 2\,\text{ms}$; for **(b)**, $\tau_a = 10\,\text{ms}$, $\tau_y = 2\,\text{ms}$; for **(c)**, $\tau_a = 2\,\text{ms}$, $\tau_y = 10\,\text{ms}$.

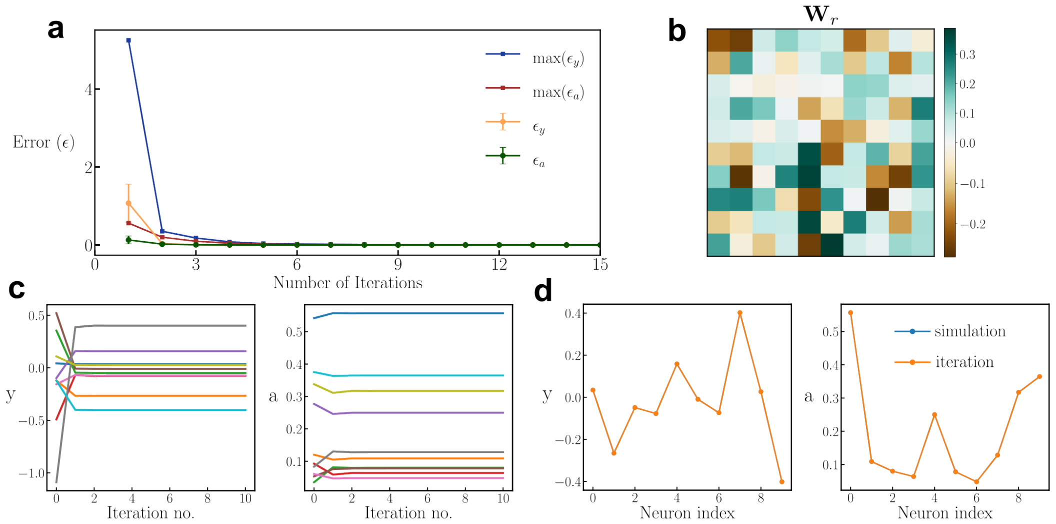

Figure 4: **Fast convergence of the iterative algorithm.** The results are for 20-dimensional ORGaNICs (10 $\mathbf{y}$ and 10 $\mathbf{a}$ neurons) with random parameters and inputs with the additional constraint of the maximum singular value of $\mathbf{W}_r$ equal to 1 and $||\mathbf{z}|| < 1$. **(a)**, Mean (with error bars representing 1-sigma S.D.) and maximum errors (ϵ) as a function of number of iterations. ϵ is calculated as the norm of the difference between the true solution (found by simulation starting with random initialization) and the iteration solution. **(b)**, An example of a randomly sampled $\mathbf{W}_r$. **(c)**, Steady-state approximation as a function of iteration number. Different lines represent different neurons. **(d)**, Overlap between the iteration solution (after 15 iterations) and the true solution.

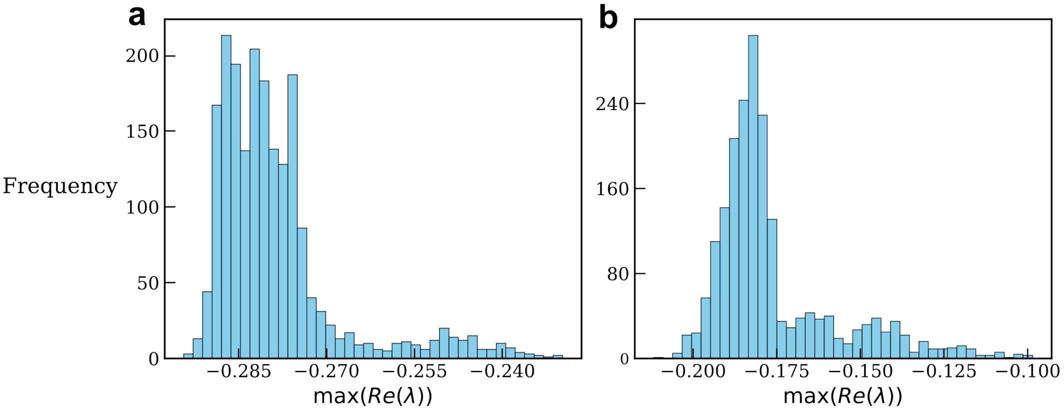

Figure 5: **Histogram for the eigenvalue with the largest real part**. We train two-layer ORGaNICs ($\tau_a = \tau_y = 2\,\text{ms}$) with a static MNIST input where $\mathbf{W}_r$ is constrained to have a maximum singular value of 1. We plot the histogram of eigenvalues of the Jacobian matrix with the largest real part, for inputs from the test set. We find that all the eigenvalues of the Jacobian have negative real parts, implying asymptotic stability. **(a)**, histogram for the first layer. **(b)**, histogram for the second layer. Note that since this is implemented in a feedforward manner, this is a cascading system with no feedback, hence we can perform the stability analysis of the two layers independently.

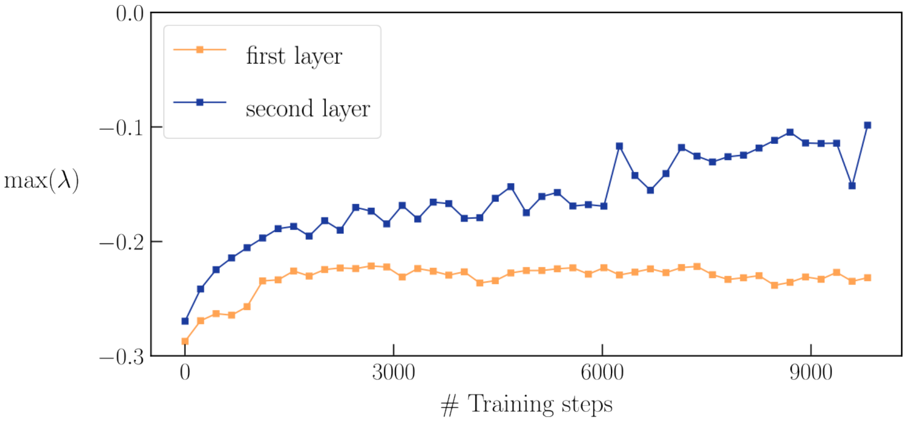

Figure 6: **Eigenvalue with the largest real part while training on static input (MNIST) classification task.** This plot shows the largest real part of eigenvalues across all test samples as training progresses. The fact that the largest real part consistently remains below zero indicates that the system maintains stability throughout training.

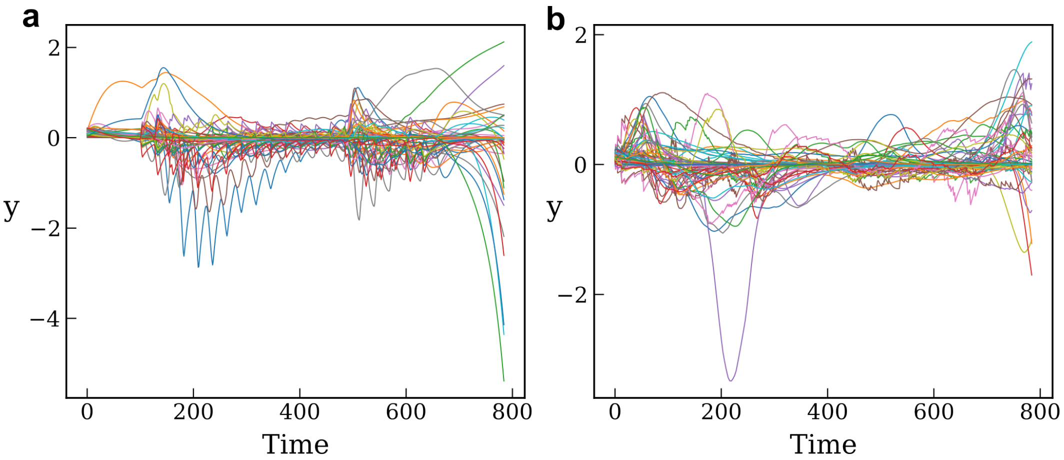

Figure 7: **Trajectories of the hidden states (y).** This plot shows the dynamics of the hidden state as the input is being presented sequentially. We train ORGaNICs (128 units) as an RNN on **(a)**, unpermuted sequential MNIST and **(b)**, permuted sequential MNIST. The inputs are picked randomly from the test set. The hidden state trajectory remains bounded, indicating stability.
