# Supplementary material for: Unconditional stability of a recurrent neural circuit implementing divisive normalization
Source: ArXiv. 2024 Sep 27:arXiv:2409.18946v1. Preprint. [Version 1] (PMC11469413)
Supplement: Supplementary file 1 [file NIHPP2409.18946V1-supplement-1.pdf]

A Derivation of the ORGaNICs circuit

Here, we derive a generalized 2-neuron type (excitatory and inhibitory) ORGaNICs model for a high dimensional input. The system presented in Eq. 1 is a special case of this generalized model where $p = 2$ and $\mathbf{a}^+ = \sqrt{\lfloor \mathbf{a} \rfloor}$. Assuming $\mathbf{W}$ is the normalization weight matrix, and $\mathbf{z}$ is the input drive, we can write the normalization equations for principal neurons with complementary receptive fields as,

$$\mathbf{y}_s^+ = \frac{\lfloor \mathbf{z} \rfloor^p}{\boldsymbol{\sigma}^p + \mathbf{W}\left(\lfloor \mathbf{z} \rfloor^p + \lfloor -\mathbf{z} \rfloor^p\right)}; \quad \mathbf{y}_s^- = \frac{\lfloor -\mathbf{z} \rfloor^p}{\boldsymbol{\sigma}^p + \mathbf{W}\left(\lfloor \mathbf{z} \rfloor^p + \lfloor -\mathbf{z} \rfloor^p\right)} \tag{17}$$

Note that typically the exponent of the input $p \sim 2$ for cortical neurons. $\lfloor \mathbf{z} \rfloor^p$ and $\lfloor -\mathbf{z} \rfloor^p$ represent the contribution of neurons with complementary receptive fields to the normalization pool. Mathematically, we have, $\lfloor \mathbf{z} \rfloor^p + \lfloor -\mathbf{z} \rfloor^p = |\mathbf{z}|^p$.

Here, we derive, for a general p, the dynamical equations that have the fixed point defined by the equation above. First, it is important to distinguish between the membrane potentials and the firing rates of neurons. The coarse (low-pass filtered) membrane potential of a given type of neuron is denoted by the vector, $\mathbf{y}, \mathbf{a}$, with the corresponding firing rates of $\mathbf{y}^+ (\mathbf{y}^-), \mathbf{a}^+$. The instantaneous firing rates of the neurons are related to the corresponding coarse membrane potential by applying rectification, denoted by $\lfloor . \rfloor$, and a power law (sub/supra-linear) activation for different types of neurons [84–86]. Therefore, for a set of membrane potentials $\mathbf{x}$ the instantaneous firing rates $\mathbf{x}^+$ are given by $\lfloor \mathbf{x} \rfloor^\alpha$. Specifically for principal neurons, we have, $\mathbf{y}^+ = \lfloor \mathbf{y} \rfloor^p$ and $\mathbf{y}^- = \lfloor -\mathbf{y} \rfloor^p$. Combining the firing of principal neurons with the complementary receptive fields, $\mathbf{y}^+$ and $\mathbf{y}^-$, Eq. 17 can be alternatively written as,

$$|\mathbf{y}_s|^p = \mathbf{y}_s^+ + \mathbf{y}_s^- = \frac{|\mathbf{z}|^p}{\boldsymbol{\sigma}^p + \mathbf{W}\,|\mathbf{z}|^p} \tag{18}$$

Now for the principal neuron y_j receiving an input drive z_j, we can rewrite the normalization equation for each neuron as,

$$|y_j^s|^p = \frac{|z_j|^p}{\sigma_j^p + \sum_k W_{jk} |z_k|^p} \tag{19}$$

In the ORGaNICs paradigm [46], the steady-state activity of the principal neurons (single equation for complementary receptive fields) is a weighted sum of input drive and recurrent drive,

$$\tau_{y_j} \frac{\mathrm{d}y_j}{\mathrm{d}t} = -y_j + \underbrace{b_j z_j}_{\text{Weighted input drive}} + \underbrace{\left(1 - a_j^+\right) \sum_k w_{rjk} \left((y_k^+)^{1/p} - (y_k^-)^{1/p}\right)}_{\text{Weighted recurrent drive}} \tag{20}$$

Here w_r are the weights of the recurrent weight matrix $\mathbf{W}_r$ encoding the recurrent/lateral connections between the principal neurons $\mathbf{y}$; y_k^+ is the firing rate of the principal neuron k, given by applying the following nonlinearity on the membrane potential: $y_k^+ = \lfloor y_k \rfloor^p$ and $y_k^- = \lfloor -y_k \rfloor^p$ is the firing rate of the complementary principal neuron k. b_j is the gain to the input drive z_j which simulates attention (realized via gain modulation). $(1 - a_j^+)$ is the gain to the recurrent drive which controls the recurrent amplification.

Now, we find the dynamics of the inhibitory neurons a_j with firing rates a_j^+, that yield stable dynamics with the fixed point given by Eq. 17. For simplicity, we assume that the recurrent weight matrix $\mathbf{W}_r = \mathbf{I}$. Also, note that the following identity holds: $(y_k^+)^{1/p} - (y_k^-)^{1/p} = \lfloor y_k \rfloor - \lfloor -y_k \rfloor = y_k$. Therefore, Eq.20 can be simplified as,

$$\tau_{y_j} \frac{\mathrm{d}y_j}{\mathrm{d}t} = -y_j + b_j z_j + \left(1 - a_j^+\right) y_j \tag{21}$$

At steady-state, the fixed-points (y_j^s, a_j^s), satisfy the following relationship,

$$a_j^{s+} y_j^s = b_j z_j \tag{22}$$

Taking modulus and raising both sides to power p, we get,

$$\left| a_j^{s+} y_j^s \right|^p = |b_j z_j|^p \tag{23}$$

or in vector form,

$$\begin{aligned} \left|\mathbf{a}_s^+ \odot \mathbf{y}_s\right|^p &= |\mathbf{b} \odot \mathbf{z}|^p \\ \left(\mathbf{a}_s^+\right)^p \odot |\mathbf{y}_s|^p &= \mathbf{b}^p \odot |\mathbf{z}|^p \end{aligned} \tag{24}$$

From Eq. 18, we know that

$$|\mathbf{z}|^p = \boldsymbol{\sigma}^p \odot |\mathbf{y}_s|^p + |\mathbf{y}_s|^p \odot (\mathbf{W}\,|\mathbf{z}|^p) \tag{25}$$

Since we can write the element-wise product between two vectors $\mathbf{x}_1$ and $\mathbf{x}_2$, $\mathbf{x}_1 \odot \mathbf{x}_2 = \mathbf{x}_2 \odot \mathbf{x}_1 = \mathbf{D}(\mathbf{x}_1)\mathbf{x}_2$. Where $\mathbf{D}(\mathbf{x}_1)$ is a diagonal matrix with elements of the vector $\mathbf{x}_1$ on the diagonal. Therefore, using this fact we can rewrite the equation above as,

$$\begin{aligned} |\mathbf{z}|^p &= \boldsymbol{\sigma}^p \odot |\mathbf{y}_s|^p + \mathbf{D}\left(|\mathbf{y}_s|^p\right)\left(\mathbf{W}\,|\mathbf{z}|^p\right) \\ |\mathbf{z}|^p &= \boldsymbol{\sigma}^p \odot |\mathbf{y}_s|^p + \mathbf{D}\left(|\mathbf{y}_s|^p\right)\mathbf{W}\,|\mathbf{z}|^p \\ \left[\mathbf{I} - \mathbf{D}\left(|\mathbf{y}_s|^p\right)\mathbf{W}\right]|\mathbf{z}|^p &= \boldsymbol{\sigma}^p \odot |\mathbf{y}_s|^p \\ |\mathbf{z}|^p &= \left[\mathbf{I} - \mathbf{D}\left(|\mathbf{y}_s|^p\right)\mathbf{W}\right]^{-1}\left(\boldsymbol{\sigma}^p \odot |\mathbf{y}_s|^p\right) \end{aligned} \tag{26}$$

Substituting the expression for $|\mathbf{z}|^p$ into Eq. 24, we get,

$$\left(\mathbf{a}_s^+\right)^p \odot |\mathbf{y}_s|^p = \mathbf{b}^p \odot \left(\left[\mathbf{I} - \mathbf{D}\left(|\mathbf{y}_s|^p\right)\mathbf{W}\right]^{-1}\left(\boldsymbol{\sigma}^p \odot |\mathbf{y}_s|^p\right)\right) \tag{27}$$

Now, we simplify this equation,

$$\begin{aligned} \left[\mathbf{I} - \mathbf{D}\left(|\mathbf{y}_s|^p\right)\mathbf{W}\right]\left(\frac{\left(\mathbf{a}_s^+\right)^p \odot |\mathbf{y}_s|^p}{\mathbf{b}^p}\right) &= \boldsymbol{\sigma}^p \odot |\mathbf{y}_s|^p \\ \mathbf{D}\left(1/|\mathbf{y}_s|^p\right)\left[\mathbf{I} - \mathbf{D}\left(|\mathbf{y}_s|^p\right)\mathbf{W}\right]\left(\frac{\left(\mathbf{a}_s^+\right)^p \odot |\mathbf{y}_s|^p}{\mathbf{b}^p}\right) &= \mathbf{D}\left(1/|\mathbf{y}_s|^p\right)\left(\boldsymbol{\sigma}^p \odot |\mathbf{y}_s|^p\right) \\ \frac{\left(\mathbf{a}_s^+\right)^p}{\mathbf{b}^p} - \mathbf{W}\left(\frac{\left(\mathbf{a}_s^+\right)^p \odot |\mathbf{y}_s|^p}{\mathbf{b}^p}\right) &= \boldsymbol{\sigma}^p \end{aligned} \tag{28}$$

Now we assume that all of the entries of $\mathbf{b}$ are equal to a constant b_0, therefore, we have,

$$\begin{aligned} \left(\mathbf{a}_s^+\right)^p - \mathbf{W}\left(\left(\mathbf{a}_s^+\right)^p \odot |\mathbf{y}_s|^p\right) &= b_0^p \boldsymbol{\sigma}^p \\ \left(\mathbf{a}_s^+\right)^p &= b_0^p \boldsymbol{\sigma}^p + \mathbf{W}\left(\left(\mathbf{a}_s^+\right)^p \odot |\mathbf{y}_s|^p\right) \end{aligned} \tag{29}$$

Element-wise multiplying both sides by $\mathbf{a}_s / \left(\mathbf{a}_s^+\right)^p$ on the left, we get,

$$\mathbf{0} = -\mathbf{a}_s + \frac{\mathbf{a}_s}{\left(\mathbf{a}_s^+\right)^p} \odot \left[b_0^p \boldsymbol{\sigma}^p + \mathbf{W}\left(\left(\mathbf{a}_s^+\right)^p \odot |\mathbf{y}_s|^p\right)\right] \tag{30}$$

This equation is true at the steady-state, but it is also in a form similar to the of $\mathbf{y}$ such that we have a weighted input drive and weighted recurrent drive, therefore, we propose the following dynamical equation for $\mathbf{a}$,

$$\boldsymbol{\tau}_a \odot \dot{\mathbf{a}} = -\mathbf{a} + \frac{\mathbf{a}}{\left(\mathbf{a}^+\right)^p} \odot \left[b_0^p \boldsymbol{\sigma}^p + \mathbf{W}\left(\left(\mathbf{a}^+\right)^p \odot |\mathbf{y}|^p\right)\right] \tag{31}$$

This equation naturally follows Eq. 30 at steady-state and thus also follows the normalization equation Eq. 18. Note that the equation above is true for any choice of $\mathbf{a}^+$, but cortical neurons have been experimentally observed to have an exponent close to $p = 2$, i.e., $\mathbf{y}^+ = \lfloor \mathbf{y} \rfloor^2$ and $\mathbf{y}^- = \lfloor -\mathbf{y} \rfloor^2$. Therefore, we get a particularly simple form of the equations when $p = 2$ and $\mathbf{a}^+ = \sqrt{\lfloor \mathbf{a} \rfloor}$. In this case, the equation for $\mathbf{a}$ is given by,

$$\boldsymbol{\tau}_a \odot \dot{\mathbf{a}} = -\mathbf{a} + \left[b_0^2 \boldsymbol{\sigma}^2 + \mathbf{W}\left(\left(\mathbf{a}^+\right)^2 \odot \mathbf{y}^2\right)\right] \tag{32}$$

We reintroduce the recurrent weight matrix $\mathbf{W}_r$ and to simulate the effect of attention by gain modulation, we define different gains for $\mathbf{y}$ and $\mathbf{a}$ neurons. Additionally, replacing $\mathbf{y}^2 \to \mathbf{y}^+ + \mathbf{y}^-$ and $\mathbf{y} \to \sqrt{\mathbf{y}^+} - \sqrt{\mathbf{y}^-}$ yields the dynamical system that we analyze,

$$\begin{aligned} \boldsymbol{\tau}_y \odot \dot{\mathbf{y}} &= -\mathbf{y} + \mathbf{b} \odot \mathbf{z} + \left(\mathbf{1} - \mathbf{a}^+\right) \odot \mathbf{W}_r \left(\sqrt{\mathbf{y}^+} - \sqrt{\mathbf{y}^-}\right) \\ \boldsymbol{\tau}_a \odot \dot{\mathbf{a}} &= -\mathbf{a} + \mathbf{b}_0^2 \odot \boldsymbol{\sigma}^2 + \mathbf{W}\,\left(\left(\mathbf{y}^+ + \mathbf{y}^-\right) \odot \mathbf{a}^{+2}\right) \end{aligned} \tag{33}$$

Finally, in the most general form, i.e., any choice of p and nonlinearity for $\mathbf{a}^+$, the dynamical system is given by,

$$\begin{aligned}
\boldsymbol{\tau}_y \odot \dot{\mathbf{y}} &= -\mathbf{y} + \mathbf{b} \odot \mathbf{z} + \left(\mathbf{1} - \mathbf{a}^+\right) \odot \mathbf{W}_r \left(\left(\mathbf{y}^+\right)^{1/p} - \left(\mathbf{y}^-\right)^{1/p}\right) \\
\boldsymbol{\tau}_a \odot \dot{\mathbf{a}} &= -\mathbf{a} + \frac{\mathbf{a}}{\left(\mathbf{a}^+\right)^p} \odot \left[\mathbf{b}_0^p \odot \boldsymbol{\sigma}^p + \mathbf{W}\left(\left(\mathbf{y}^+ + \mathbf{y}^-\right) \odot \left(\mathbf{a}^+\right)^p\right)\right]
\end{aligned} \tag{34}$$

B Stability theorem

Theorem B.1. *Consider a system of linear differential equations with constant coefficients of the form,*

$$\mathbf{I}\ddot{\mathbf{x}} + \mathbf{B}\dot{\mathbf{x}} + \mathbf{K}\mathbf{x} = \mathbf{0} \tag{35}$$

where $\mathbf{B} \in \mathbb{R}^{n \times n}$ and $\mathbf{K} \in \mathbb{R}^{n \times n}$ is a positive diagonal matrix, therefore, $\mathbf{K} \succ 0$. The dynamical system is globally asymptotically stable if $\mathbf{B}$ is Lyapunov diagonally stable.

Proof. The stability of the system is defined by solving the following associated quadratic eigenvalue problem,

$$\mathbb{L}(\lambda) = \det(\lambda^2 \mathbf{I} + \mathbf{B}\lambda + \mathbf{K}) \tag{36}$$

The spectrum of $\mathbb{L}(\lambda)$, i.e., $\{\lambda \in \mathbf{C} : \det(\mathbb{L}(\lambda)) = 0\}$ are also known as the eigenvalues of the system. The system defined by Eq. 35 is globally asymptotically stable if all of the eigenvalues have negative real parts.

We take the direct Lyapunov approach to prove the stability of the linear system. We write Eq. 35 in the matrix form as $\dot{\mathbf{z}} = \mathbf{J}\mathbf{z}$, where,

$$\mathbf{z} = \begin{bmatrix} \mathbf{x} \\ \dot{\mathbf{x}} \end{bmatrix} \quad \text{and} \quad \mathbf{J} = \begin{bmatrix} \mathbf{0} & \mathbf{I} \\ -\mathbf{K} & -\mathbf{B} \end{bmatrix} \tag{37}$$

We will first prove the Lyapunov stability ($\mathrm{Re}(\lambda_{\mathbf{J}}) \leq 0$) of this system to find the appropriate block diagonal matrices and then we will prove global asymptotic stability. To prove Lyapunov stability (Appendix B.2), we propose a Lyapunov function $V(\mathbf{z}) = \mathbf{z}^\top \mathbf{P} \mathbf{z}$, where $\mathbf{P}$ is a block positive definite matrix defined as follows,

$$\mathbf{P} = \begin{bmatrix} \mathbf{A} & \mathbf{0} \\ \mathbf{0} & \mathbf{T} \end{bmatrix} \tag{38}$$

where $\mathbf{T} \in \mathbb{R}^{n \times n}$ is a positive diagonal matrix such that $\mathbf{TB} + \mathbf{B}^\top \mathbf{T} \succ 0$ (notation for positive definite matrix) and $\mathbf{A} \in \mathbb{R}^{n \times n}$ a flexible symmetric positive definite matrix that we'll find using the second Lyapunov criteria. Note that such a matrix $\mathbf{T}$ exits since $\mathbf{B}$ is defined to be Lyapunov diagonally stable. It can be easily seen that $\mathbf{P} \succ 0$ using the first criteria in Section B.1 since $\mathbf{A} \succ 0$ and $\mathbf{T} \succ 0$. Additionally, since $\mathbf{T} \succ 0$, it is invertible.

Now, for Lyapunov stability, we need $\dot{V}(\mathbf{z}) = \mathbf{z}^\top \left(\mathbf{PJ} + \mathbf{J}^\top \mathbf{P}\right) \mathbf{z} \leq 0$. Therefore, we find $\mathbf{A}$ such that $\mathbf{Q} = -\left(\mathbf{PJ} + \mathbf{J}^\top \mathbf{P}\right)$ is positive semi-definite.

$$\begin{aligned}
\mathbf{Q} &= -\left(\mathbf{PJ} + \mathbf{J}^\top \mathbf{P}\right) \\
&= -\begin{bmatrix} \mathbf{A} & \mathbf{0} \\ \mathbf{0} & \mathbf{T} \end{bmatrix} \begin{bmatrix} \mathbf{0} & \mathbf{I} \\ -\mathbf{K} & -\mathbf{B} \end{bmatrix} - \begin{bmatrix} \mathbf{0} & -\mathbf{K} \\ \mathbf{I} & -\mathbf{B}^\top \end{bmatrix} \begin{bmatrix} \mathbf{A} & \mathbf{0} \\ \mathbf{0} & \mathbf{T} \end{bmatrix} \\
&= \begin{bmatrix} \mathbf{0} & \mathbf{KT} - \mathbf{A} \\ \mathbf{TK} - \mathbf{A} & \mathbf{TB} + \mathbf{B}^\top \mathbf{T} \end{bmatrix}
\end{aligned} \tag{39}$$

We want to define $\mathbf{A} \succ 0$, such that $\mathbf{Q} \succeq 0$. Using the second criteria from Section B.1, we need $\mathbf{TB} + \mathbf{B}^\top \mathbf{T} \succ 0$ and $-(\mathbf{KT} - \mathbf{A})(\mathbf{TB} + \mathbf{B}^\top \mathbf{T})^{-1}(\mathbf{TK} - \mathbf{A}) \succeq 0$. The first condition is satisfied by the definition of $\mathbf{T}$. For the second condition to be satisfied, an obvious candidate for $\mathbf{A}$ is $\mathbf{TK}$. Note that both $\mathbf{T}$ and $\mathbf{K}$ are positive definite and diagonal, therefore they commute ($\mathbf{KT} = \mathbf{TK}$) and $\mathbf{A}$ is symmetric and positive definite. When $\mathbf{A} = \mathbf{TK}$, the LHS of the second condition becomes $\mathbf{0} \succeq 0$. Therefore, the system is Lyapunov stable.

Now, we prove the global asymptotic stability of the system by again using the direct Lyapunov approach. We propose the same form of the Lyapunov function as before, i.e., $V(\mathbf{z}) = \mathbf{z}^\top \mathbf{P} \mathbf{z}$, where

$\mathbf{P}$ is a positive definite matrix $\mathbf{P}$. But for global asymptotic stability, we need a more stringent condition on the Lyapunov function, $\dot{V}(\mathbf{z}) = \mathbf{z}^\top \left(\mathbf{PJ} + \mathbf{J}^\top\mathbf{P}\right) \mathbf{z} < 0$. Drawing inspiration from the previous exercise, we consider the following form of the matrix $\mathbf{P}$,

$$\mathbf{P} = \begin{bmatrix} \mathbf{TK} & \epsilon\mathbf{I} \\ \epsilon\mathbf{I} & \mathbf{T} \end{bmatrix} \tag{40}$$

Here, $\epsilon > 0$ is a scalar whose magnitude is to be determined based on the Lyapunov criteria for asymptotic stability. First, we need $\mathbf{P} \succ 0$. Applying the first criteria from Section B.1, we want ϵ to satisfy, $\mathbf{TK} - \epsilon^2\mathbf{T}^{-1} \succ 0$. Second, for $\dot{V}(\mathbf{z}) < 0$, we want $\mathbf{Q} = -\left(\mathbf{PJ} + \mathbf{J}^\top\mathbf{P}\right)$ to be positive definite. $\mathbf{Q}$ is given by,

$$\begin{aligned} \mathbf{Q} &= -\begin{bmatrix} \mathbf{TK} & \epsilon\mathbf{I} \\ \epsilon\mathbf{I} & \mathbf{T} \end{bmatrix} \begin{bmatrix} \mathbf{0} & \mathbf{I} \\ -\mathbf{K} & -\mathbf{B} \end{bmatrix} - \begin{bmatrix} \mathbf{0} & -\mathbf{K} \\ \mathbf{I} & -\mathbf{B}^\top \end{bmatrix} \begin{bmatrix} \mathbf{TK} & \epsilon\mathbf{I} \\ \epsilon\mathbf{I} & \mathbf{T} \end{bmatrix} \\ &= \begin{bmatrix} 2\epsilon\mathbf{K} & \epsilon\mathbf{B} \\ \epsilon\mathbf{B}^\top & \mathbf{TB} + \mathbf{B}^\top\mathbf{T} - 2\epsilon\mathbf{I} \end{bmatrix} \end{aligned} \tag{41}$$

We again apply the first criteria from Section B.1. $\mathbf{Q} \succ 0$ if and only if $\mathbf{TB} + \mathbf{B}^\top\mathbf{T} - 2\epsilon\mathbf{I} \succ 0$ and $2\epsilon\mathbf{K} - \epsilon^2\mathbf{B}\left(\mathbf{TB} + \mathbf{B}^\top\mathbf{T} - 2\epsilon\mathbf{I}\right)^{-1}\mathbf{B}^\top \succ 0$. To simplify notation we replace $\mathbf{TB} + \mathbf{B}^\top\mathbf{T}$ with a positive definite matrix $\mathbf{M}$. Therefore, we have to prove that there exists an $\epsilon > 0$ which satisfies the following conditions,

- $\mathbf{TK} - \epsilon^2\mathbf{T}^{-1} \succ 0$
- $\mathbf{M} - 2\epsilon\mathbf{I} \succ 0$
- $2\mathbf{K} - \epsilon\mathbf{B}\left(\mathbf{M} - 2\epsilon\mathbf{I}\right)^{-1}\mathbf{B}^\top \succ 0$

Assuming t_i and k_i to be the diagonal values of the positive diagonal matrices $\mathbf{T}$ and $\mathbf{K}$, we get the following two conditions, $\epsilon < \min\left(t_i\sqrt{k_i}\right)$ and $\epsilon < \alpha/2$, where α is the smallest eigenvalue of $\mathbf{M}$, or $\alpha = \min\left(\lambda_{\mathbf{M}}\right)$.

Now, for the third condition, we will use a bunch of facts about positive definite matrices which are all listed in [87]. We first consider the following matrix inequality, $\mathbf{M} \succeq \alpha\mathbf{I}$. This notation is equivalent to saying that $\mathbf{M} - \alpha\mathbf{I}$ is positive semi-definite or $\mathbf{M} - \alpha\mathbf{I} \succeq 0$. Therefore, we have,

$$\mathbf{M} - 2\epsilon\mathbf{I} \succeq (\alpha - 2\epsilon)\mathbf{I} \tag{42}$$

Assuming, ϵ is small enough such that the matrices on LHS and RHS are positive definite, we have,

$$\frac{1}{\alpha - 2\epsilon}\mathbf{I} \succeq \left(\mathbf{M} - 2\epsilon\mathbf{I}\right)^{-1} \tag{43}$$

Since $\mathbf{B}$ is nonsingular, it is full rank, therefore, we have,

$$\frac{1}{\alpha - 2\epsilon}\mathbf{BB}^\top \succeq \mathbf{B}\left(\mathbf{M} - 2\epsilon\mathbf{I}\right)^{-1}\mathbf{B}^\top \tag{44}$$

Multiplying both sides by ϵ, we get,

$$\frac{\epsilon}{\alpha - 2\epsilon}\mathbf{BB}^\top \succeq \epsilon\mathbf{B}\left(\mathbf{M} - 2\epsilon\mathbf{I}\right)^{-1}\mathbf{B}^\top \tag{45}$$

Notice that $\mathbf{BB}^\top$ is a positive definite matrix. Let β be the maximum eigenvalue of $\mathbf{BB}^\top$, or $\beta = \max\left(\lambda_{\mathbf{BB}^\top}\right)$. Therefore, we can add an upper-bound matrix to the inequality as follows,

$$\frac{\epsilon\beta}{\alpha - 2\epsilon}\mathbf{I} \succeq \frac{\epsilon}{\alpha - 2\epsilon}\mathbf{BB}^\top \succeq \epsilon\mathbf{B}\left(\mathbf{M} - 2\epsilon\mathbf{I}\right)^{-1}\mathbf{B}^\top \tag{46}$$

Now, we find ϵ such that,

$$2\mathbf{K} \succ \frac{\epsilon\beta}{\alpha - 2\epsilon}\mathbf{I} \tag{47}$$

The range of values for which the above inequality is true is,

$$\epsilon < \min\left(\frac{2k_i\alpha}{\beta + 4k_i}\right) \tag{48}$$

If ϵ satisfies the inequality above, we have,

$$2\mathbf{K} \succ \frac{\epsilon\beta}{\alpha - 2\epsilon}\mathbf{I} \succeq \frac{\epsilon}{\alpha - 2\epsilon}\mathbf{B}\mathbf{B}^\top \succeq \epsilon\mathbf{B}\left(\mathbf{M} - 2\epsilon\mathbf{I}\right)^{-1}\mathbf{B}^\top \tag{49}$$

Therefore, we have, $2\mathbf{K} \succ \epsilon\mathbf{B}\left(\mathbf{M} - 2\epsilon\mathbf{I}\right)^{-1}\mathbf{B}^\top$ and the third condition required for ϵ is satisfied. This implies that there exists a range of ϵ, given by,

$$0 < \epsilon < \min\left\{\min\left(t_i\sqrt{k_i}\right),\ \frac{\alpha}{2},\ \min\left(\frac{2k_i\alpha}{\beta + 4k_i}\right)\right\} \tag{50}$$

for which

$$\mathbf{P} = \begin{bmatrix}\mathbf{TK} & \epsilon\mathbf{I} \\ \epsilon\mathbf{I} & \mathbf{T}\end{bmatrix} \tag{51}$$

is a valid Lyapunov function for asymptotic stability. Therefore, the dynamical system is globally asymptotically stable.

□

B.1 Positive definite block matrices (Schur complement)

For a symmetric block matrix of the form,

$$\mathbf{P} = \begin{bmatrix}\mathbf{A} & \mathbf{B} \\ \mathbf{B}^\top & \mathbf{C}\end{bmatrix} \tag{52}$$

with $\mathbf{A} = \mathbf{A}^\top$ and $\mathbf{C} = \mathbf{C}^\top$. If $\mathbf{C}$ is invertible the following two properties hold, [88],

- $\mathbf{P} \succ 0$ if and only if $\mathbf{C} \succ 0$ and $\mathbf{A} - \mathbf{B}\mathbf{C}^{-1}\mathbf{B}^\top \succ 0$.
- If $\mathbf{C} \succ 0$, then $\mathbf{P} \succeq 0$ if and only if $\mathbf{A} - \mathbf{B}\mathbf{C}^{-1}\mathbf{B}^\top \succeq 0$.

B.2 Lyapunov stability criteria

Consider a non-linear autonomous dynamical system defined as $\dot{\mathbf{x}} = \mathbf{f}(\mathbf{x})$, with a point of equilibrium at $\mathbf{x} = \mathbf{0}$. Where $\mathbf{x} \in \mathcal{D} \subseteq \mathbb{R}^n$ is the system state vector and $\mathbf{f}(\mathbf{x}) : \mathcal{D} \to \mathbb{R}^n$ is a continuous vector field on $\mathcal{D}$ (contains origin). The dynamical system is called Lyapunov stable if there exists a real scalar function $V(\mathbf{x}) : \mathbb{R}^n \to \mathbb{R}$, also known as the Lyapunov function, such that it satisfies the following conditions,

- $V(\mathbf{x}) = 0$, if and only if $\mathbf{x} = \mathbf{0}$.
- $V(\mathbf{x}) > 0$, if and only if $\mathbf{x} \neq \mathbf{0}$.
- $\dot{V}(\mathbf{x}) \leq 0$, $\forall \mathbf{x} \neq \mathbf{0}$. Note that for asymptotic stability, we require the strict inequality $\dot{V}(\mathbf{x}) < 0$.

For a linear dynamical system of the form $\dot{\mathbf{x}} = \mathbf{J}\mathbf{x}$, where $\mathbf{J} \in \mathbb{R}^{n\times n}$, with a point of equilibrium at $\mathbf{x} = \mathbf{0}$. Consider a Lyapunov function $V(\mathbf{x})$ of the form $\mathbf{x}^\top\mathbf{P}\mathbf{x}$, such that $\mathbf{P} \succ 0$. By the definition of a positive definite matrix, it satisfies the first two conditions, namely, $V(\mathbf{x}) = \mathbf{x}^\top\mathbf{P}\mathbf{x} = 0$ when $\mathbf{x} = \mathbf{0}$ and $V(\mathbf{x}) = \mathbf{x}^\top\mathbf{P}\mathbf{x} > 0$ when $x \neq \mathbf{0}$. For the third condition, consider $\dot{V}(\mathbf{x})$,

$$\begin{aligned}\dot{V}(\mathbf{x}) &= \frac{\mathrm{d}}{\mathrm{d}t}V(\mathbf{x}) \\ &= \frac{\mathrm{d}}{\mathrm{d}t}\mathbf{x}^\top\mathbf{P}\mathbf{x} \\ &= \mathbf{x}^\top\mathbf{P}\dot{\mathbf{x}} + \dot{\mathbf{x}}^\top\mathbf{P}\mathbf{x} \\ &= \mathbf{x}^\top\left(\mathbf{P}\mathbf{J} + \mathbf{J}^\top\mathbf{P}\right)\mathbf{x}\end{aligned} \tag{53}$$

For stability, We need $\dot{V}(\mathbf{x}) = \mathbf{x}^\top\left(\mathbf{P}\mathbf{J} + \mathbf{J}^\top\mathbf{P}\right)\mathbf{x} \leq 0$. This is satisfied when the matrix $\mathbf{P}\mathbf{J} + \mathbf{J}^\top\mathbf{P} \preceq 0$. In summary, a linear dynamical system $\dot{\mathbf{x}} = \mathbf{J}\mathbf{x}$ is Lyapunov stable if there exists a positive definite matrix $\mathbf{P}$, such that $\mathbf{P}\mathbf{J} + \mathbf{J}^\top\mathbf{P}$ is negative semi-definite.

C Analytical eigenvalue for fully normalized circuit

Here we show that when all of the normalization weights in the system are equal, to value α, and the various parameters are scalars, i.e., $\boldsymbol{\tau}_y = \tau_y \mathbf{1}$, $\boldsymbol{\tau}_a = \tau_a \mathbf{1}$, $\mathbf{b}_0 = b_0 \mathbf{1}$ and $\boldsymbol{\sigma} = \sigma \mathbf{1}$, we can derive an analytical expression for all of the eigenvalues. Considering these assumptions, we can break the determinant in Eq. 8 into a diagonal and non-diagonal part as

$$\det(\mathbf{J} - \lambda \mathbf{I}) = \det\left(\lambda^2 \mathbf{I} + \lambda \left[\frac{\mathbf{I}}{\tau_a} + \frac{\mathbf{D}\left(\sqrt{\mathbf{a}_s}\right)}{\tau_y} \right] + \frac{\mathbf{D}\left(\sqrt{\mathbf{a}_s}\right)}{\tau_y \tau_a} - \lambda \frac{\mathbf{W}\mathbf{D}\left(\mathbf{y}_s{}^2\right)}{\tau_a} \right) \tag{54}$$

Consider the non-diagonal part of the matrix in the determinant, $(\lambda/\tau_a)\mathbf{W}\mathbf{D}\left(\mathbf{y}_s^2\right)$. Since $\mathbf{W}$ is a matrix with all entries equal to a positive constant, α, it is rank 1. Therefore, it can be written as the following outer product,

$$\mathbf{W} = \alpha \begin{bmatrix} 1 \\ 1 \\ \vdots \\ 1 \end{bmatrix} \begin{bmatrix} 1 & 1 & \dots & 1 \end{bmatrix} \tag{55}$$

Therefore, the non-diagonal part of the matrix can be written as,

$$\frac{\lambda}{\tau_a} \mathbf{W}\,\mathbf{D}\left(\frac{\mathbf{b}^2 \odot \mathbf{z}^2}{\sigma^2 b_0^2 \mathbf{1} + \mathbf{W}\left(\mathbf{b}^2 \odot \mathbf{z}^2\right)} \right) = \frac{\lambda \alpha}{\tau_a} \mathbf{u}\mathbf{v}^\top \tag{56}$$

where $\mathbf{u} = [1, 1, ..., 1]^\top$ and $\mathbf{v} = \left(\mathbf{b}^2 \odot \mathbf{z}^2\right) / \left(\sigma^2 b_0^2 \mathbf{1} + \mathbf{W}\left(\mathbf{b}^2 \odot \mathbf{z}^2\right)\right)$. We use the matrix determinant lemma which states that,

$$\det(\mathbf{A} - \gamma \mathbf{u}\mathbf{v}^\top) = (1 - \gamma \mathbf{v}^\top \mathbf{A}^{-1} \mathbf{u}) \det(\mathbf{A}) \tag{57}$$

The matrix $\mathbf{A}$ is given by,

$$\begin{aligned}
\mathbf{A} &= \lambda^2 \mathbf{I} + \lambda \left[\frac{\mathbf{I}}{\tau_a} + \frac{\mathbf{D}\left(\sqrt{\mathbf{a}_s}\right)}{\tau_y} \right] + \frac{\mathbf{D}\left(\sqrt{\mathbf{a}_s}\right)}{\tau_y \tau_a} \\
&= \lambda^2 \mathbf{I} + \lambda \left[\frac{\mathbf{I}}{\tau_a} + \frac{\mathbf{D}\left(\sqrt{\sigma^2 b_0^2 \mathbf{1} + \mathbf{W}\left(\mathbf{b}^2 \odot \mathbf{z}^2\right)}\right)}{\tau_y} \right] + \frac{\mathbf{D}\left(\sqrt{\sigma^2 b_0^2 \mathbf{1} + \mathbf{W}\left(\mathbf{b}^2 \odot \mathbf{z}^2\right)}\right)}{\tau_y \tau_a} \\
&= \lambda^2 \mathbf{I} + \lambda \left[\frac{\mathbf{I}}{\tau_a} + \frac{\mathbf{D}\left(\sqrt{\sigma^2 b_0^2 \mathbf{1} + \alpha ||\mathbf{b} \odot \mathbf{z}||^2}\right)}{\tau_y} \right] + \frac{\mathbf{D}\left(\sqrt{\sigma^2 b_0^2 \mathbf{1} + \alpha ||\mathbf{b} \odot \mathbf{z}||^2}\right)}{\tau_y \tau_a} \\
&= \left(\lambda^2 + \lambda \left(\frac{1}{\tau_a} + \frac{\sqrt{\sigma^2 b_0^2 + \alpha ||\mathbf{b} \odot \mathbf{z}||^2}}{\tau_y} \right) + \frac{\sqrt{\sigma^2 b_0^2 + \alpha ||\mathbf{b} \odot \mathbf{z}||^2}}{\tau_y \tau_a} \right) \mathbf{I}
\end{aligned} \tag{58}$$

Here, $||x||$, represents the Euclidean norm of x. Therefore, $\mathbf{A} = \delta \mathbf{I}$, where δ is a quadratic scalar polynomial in λ. Now using Eq. 57, we can write

$$\begin{aligned}
\det(\mathbf{J} - \lambda \mathbf{I}) &= \left(1 - \frac{\lambda \alpha}{\tau_a} \mathbf{v}^\top \left(\frac{1}{\delta} \mathbf{I} \right) \mathbf{u} \right) \delta^n \\
&= \left(\delta - \frac{\lambda}{\tau_a} \frac{\alpha ||\mathbf{b} \odot \mathbf{z}||^2}{\sigma^2 b_0^2 + \alpha ||\mathbf{b} \odot \mathbf{z}||^2} \right) \delta^{n-1}
\end{aligned} \tag{59}$$

Solving for the eigenvalues, $\det(\mathbf{J} - \lambda \mathbf{I}) = 0$, we get $2(n-1)$ repeated solutions by equation $\delta^{n-1} = 0$, where each solution is found by solving $\delta = 0$,

$$\lambda^2 + \lambda \left(\frac{1}{\tau_a} + \frac{\sqrt{\sigma^2 b_0^2 + \alpha ||\mathbf{b} \odot \mathbf{z}||^2}}{\tau_y} \right) + \frac{\sqrt{\sigma^2 b_0^2 + \alpha ||\mathbf{b} \odot \mathbf{z}||^2}}{\tau_y \tau_a} = 0 \tag{60}$$

This gives us the following strictly negative eigenvalues,

$$\lambda = -\frac{1}{\tau_a} \quad \& \quad \lambda = -\frac{\sqrt{\sigma^2 b_0^2 + \alpha ||\mathbf{b} \odot \mathbf{z}||^2}}{\tau_y} \tag{61}$$

The potentially complex eigenvalues are given by solving for zeroes of the first part of the factorized determinant,

$$\lambda^2 + \lambda \left(\frac{1}{\tau_a} \left(1 - \frac{\alpha ||\mathbf{b} \odot \mathbf{z}||^2}{\sigma^2 b_0^2 + \alpha ||\mathbf{b} \odot \mathbf{z}||^2} \right) + \frac{\sqrt{\sigma^2 b_0^2 + \alpha ||\mathbf{b} \odot \mathbf{z}||^2}}{\tau_y} \right) + \frac{\sqrt{\sigma^2 b_0^2 + \alpha ||\mathbf{b} \odot \mathbf{z}||^2}}{\tau_y \tau_a} = 0 \tag{62}$$

The eigenvalues found by solving this equation have negative real parts (as expected) for all choices of parameters since the coefficient of λ and the constant term of the quadratic equation are larger than 0 for all choices of parameters. Therefore, this solution does admit complex roots (thereby oscillations) for certain choices of the parameters which can be found by solving this quadratic equation.

D Convergent theorem

Theorem D.1. *A matrix* $\mathbf{A}$ *of the form,*

$$\mathbf{A} = \mathbf{D}(\mathbf{t})\, \mathbf{W}\, \mathbf{D}\left(\frac{\mathbf{u}}{\mathbf{v} + \mathbf{W}\mathbf{u}} \right) \tag{63}$$

is convergent, i.e., its spectral radius is less than one, if $\mathbf{W} \in \mathbb{R}^{n \times n}$ *and* $\mathbf{t}, \mathbf{u}, \mathbf{v} \in \mathbb{R}^n$ *with the additional constraints* $0 < t_i < 1$, $u_i \geq 0$, $v_i > 0$ *and* $w_{ij} \geq 0$ *for all* i, j.

Proof. Assuming the constraints mentioned in the theorem, we first notice that,

$$\mathbf{W}\, \mathbf{D}\left(\frac{\mathbf{u}}{\mathbf{v} + \mathbf{W}\mathbf{u}} \right) \tag{64}$$

is a nonnegative matrix. Further, because $\mathbf{D}(\mathbf{t})$ is a positive diagonal matrix with all entries less than 1, we notice that the following is true element-wise,

$$\mathbf{D}(\mathbf{t})\, \mathbf{W}\, \mathbf{D}\left(\frac{\mathbf{u}}{\mathbf{v} + \mathbf{W}\mathbf{u}} \right) < \mathbf{W}\, \mathbf{D}\left(\frac{\mathbf{u}}{\mathbf{v} + \mathbf{W}\mathbf{u}} \right) \tag{65}$$

We denote the spectral radius of $\mathbf{A}$, $\max\{|\lambda| : \lambda = \sigma(\mathbf{A})\}$, by $\rho(\mathbf{A})$, where $\sigma(\mathbf{A})$ is the spectrum of $\mathbf{A}$ and make use of the following inequality for the spectral radius of nonnegative matrices.,

Theorem D.2. *(Theorem 8.1.18 from [89]) Let* $\mathbf{X}$ *and* $\mathbf{Y}$ *be nonnegative matrices with spectral radius* $\rho(\mathbf{X})$ *and* $\rho(\mathbf{Y})$*, respectively. If* $\mathbf{X} \leq \mathbf{Y}$ *(*$x_{ij} \leq y_{ij}, \forall\, i, j$*), then* $\rho(\mathbf{X}) \leq \rho(\mathbf{Y})$.

Therefore, we have,

$$\rho\left(\mathbf{A}\right) \leq \rho\left(\mathbf{W}\, \mathbf{D}\left(\frac{\mathbf{u}}{\mathbf{v} + \mathbf{W}\mathbf{u}} \right) \right) \tag{66}$$

Now the elements of the matrix are given by,

$$\mathbf{W}\, \mathbf{D}\left(\frac{\mathbf{u}}{\mathbf{v} + \mathbf{W}\mathbf{u}} \right) = \begin{bmatrix} w_{11} & w_{12} & \cdots \\ w_{21} & w_{22} & \cdots \\ \vdots & \vdots & \ddots \end{bmatrix} \begin{bmatrix} \frac{u_1}{v_1 + \sum_j w_{1j} u_j} & 0 & \cdots \\ 0 & \frac{u_2}{v_2 + \sum_j w_{2j} u_j} & \cdots \\ \vdots & \vdots & \ddots \end{bmatrix} \tag{67}$$

Let s_{ij} be the i, j element of this matrix. Upon multiplication of the matrices above, we find that,

$$s_{ij} = \frac{w_{ij} u_j}{v_j + \sum_k w_{jk} u_k} \tag{68}$$

Now we use the following theorem that provides an upper bound for the spectral radius based on the entries of the matrix.

Theorem D.3. *(Theorem 8.1.26 from [89]) Let* $\mathbf{S}$ *be a nonnegative matrix. Then for any positive vector* $\mathbf{x} \in \mathbb{R}^n$ *with entries* x_j*, we have,*

$$\rho(\mathbf{S}) \leq \max_{1 \leq i \leq n} \frac{1}{x_i} \sum_{j=1}^{n} s_{ij} x_j \tag{69}$$

We pick $x_j = v_j + \sum_k w_{jk} u_k$, which is a positive vector, and apply the theorem to the matrix. Substituting s_{ij} and x_j, we get the bound for the spectral radius defined by,

$$\rho\left(\mathbf{W}\,\mathbf{D}\left(\frac{\mathbf{u}}{\mathbf{v}+\mathbf{W}\mathbf{u}}\right)\right) \leq \max_{1\leq i\leq n} \frac{1}{v_i + \sum_k w_{ik}u_k} \sum_{j=1}^{n} \frac{w_{ij}u_j}{v_j + \sum_k w_{jk}u_k} \left(v_j + \sum_k w_{jk}u_k\right) \tag{70}$$

Upon simplification, we get,

$$\rho\left(\mathbf{W}\,\mathbf{D}\left(\frac{\mathbf{u}}{\mathbf{v}+\mathbf{W}\mathbf{u}}\right)\right) \leq \max_{1\leq i\leq n} \frac{\sum_j w_{ij}u_j}{v_i + \sum_k w_{ik}u_k} \leq \max_{1\leq i\leq n} \frac{\sum_k w_{ik}u_k}{v_i + \sum_k w_{ik}u_k} < 1 \tag{71}$$

The inequality is true, because, for all i, the denominator is larger than the numerator because of the extra positive term v_i. Therefore,

$$\rho\left(\mathbf{A}\right) \leq \rho\left(\mathbf{W}\,\mathbf{D}\left(\frac{\mathbf{u}}{\mathbf{v}+\mathbf{W}\mathbf{u}}\right)\right) < 1 \tag{72}$$

and $\mathbf{A}$ is a convergent matrix. □

E Linear stability analysis of the two-dimensional model

Here we consider the dynamical stability of the 2D model containing one neuron each of y and a when the recurrent scalar, w_r can take any positive value. The dynamical system to be analyzed is given by,

$$\begin{aligned} \tau_y \dot{y} &= -y + bz + \left(1 - \sqrt{\lfloor a \rfloor}\right) w_r y \\ \tau_a \dot{a} &= -a + b_0^2\sigma^2 + wy^2\lfloor a \rfloor \end{aligned} \tag{73}$$

with a positive real constraint on the following set of parameters, $\tau_y, \tau_a, b, b_0, \sigma, w$. We first notice the symmetry in the dynamical system about $y = 0$. Replacing, $\hat{y} \to -y$, we get the mirrored dynamical system,

$$\begin{aligned} \tau_{\hat{y}} \dot{\hat{y}} &= -\hat{y} + b(-z) + \left(1 - \sqrt{\lfloor a \rfloor}\right) w_r \hat{y} \\ \tau_a \dot{a} &= -a + b_0^2\sigma^2 + w\hat{y}^2\lfloor a \rfloor \end{aligned} \tag{74}$$

These equations are the same as Eq. 73, up to a sign change in z. Therefore, we can derive analogous conditions for stability for $z < 0$ once the conditions for $z > 0$ are known.

The steady-state of Eq. 73, (y_s, a_s) satisfies the following equations,

$$y_s = bz + \left(1 - \sqrt{\lfloor a_s \rfloor}\right) w_r y_s \tag{75}$$

$$a_s = b_0^2\sigma^2 + wy_s^2\lfloor a_s \rfloor \tag{76}$$

Since the R.H.S. of the second equation is always positive, if a root exists, we have $a_s > 0$. Therefore, we can remove the rectification around a_s and look for positive solutions for a_s. Upon rearranging the terms, we get,

$$(1 - w_r + w_r\sqrt{a_s})\, y_s = bz \tag{77}$$

$$(1 - wy_s^2)\, a_s = b_0^2\sigma^2 \tag{78}$$

Substituting y_s from Eq. 77 in Eq. 78, we get the following quartic equation in $m = \sqrt{a_s}$,

$$\begin{aligned} {w_r}^2 m^4 + 2(1-w_r)w_r m^3 + &\left((1-w_r)^2 - wb^2z^2 - b_0^2\sigma^2{w_r}^2\right) m^2 \\ &- 2(1-w_r)w_r b_0^2\sigma^2 m - (1-w_r)^2 b_0^2\sigma^2 = 0 \end{aligned} \tag{79}$$

For a valid fixed point (y_s, a_s), i.e., $y_s \in \mathbb{R}$ and $a_s \in \mathbb{R}_*^+$, a necessary condition is that a_s must be positive and real, which in turn implies m must be positive and real.

Theorem E.1. *A fixed point, (y_s, a_s), is valid if and only if it satisfies $\sqrt{a_s} > 0$.*

Proof. Due to Lemma E.2, we have: a fixed point, (y_s, a_s), is valid if and only if it satisfies $m > b_0\sigma$. Further due to Lemma E.3, we have that the conditions $m > 0$ and $m > b_0\sigma$ are equivalent. Combining these two we get the statement of the theorem. $\square$

Lemma E.2. *A fixed point, (y_s, a_s), is valid if and only if it satisfies $\sqrt{a_s} > b_0\sigma$.*

Proof. $\Longrightarrow$ Given, $m = \sqrt{a_s} > b_0\sigma$, we have $a_s > b_0^2\sigma^2$, therefore, $a_s \in \mathbb{R}_*^+$; and $y_s = \sqrt{m^2 - b_0^2\sigma^2}/(m\sqrt{w})$, therefore, $y_s \in \mathbb{R}$. $\Longleftarrow$ Given $a_s \in \mathbb{R}_*^+$ and $y_s \in \mathbb{R}$, implies $m \in \mathbb{R}_*^+$. Now, Eq. 78 posits that, $m = b_0\sigma/\sqrt{1 - wy_s^2}$, therefore, $m > b_0\sigma$. $\square$

Lemma E.3. *For a positive real root, $m = \sqrt{a_s}$, to the quartic equation (Eq. 79), the condition $m > 0$ is equivalent to the condition $m > b_0\sigma$. Further, no fixed point satisfies $0 < m < b_0\sigma$.*

Proof. $\Longrightarrow$ Given $m > b_0\sigma$, we have $m > 0$ because $b_0 > 0$ and $\sigma > 0$. $\Longleftarrow$ Since m is a root of the quartic, it also satisfies Eq. 78. Therefore, we have $m = b_0\sigma/\sqrt{1 - wy_s^2}$. Since we're given $m > 0$, this implies that $0 < 1 - wy_s^2 < 1$, or $m > b_0\sigma$. Therefore, $m > 0$ is equivalent to $m > b_0\sigma$. This also implies that there exists no fixed point that satisfies $0 < m < b_0\sigma$. $\square$

Further, the Jacobian matrix at the fixed point of the dynamical system, in terms of the parameters and the fixed point is given by,

$$\mathbf{J} = \begin{bmatrix} \frac{w_r - 1 - w_r\sqrt{a_s}}{\tau_y} & -\frac{w_r y_s}{2\sqrt{a_s}\tau_y} \\ \frac{2wa_s y_s}{\tau_a} & \frac{-1 + wy_s^2}{\tau_a} \end{bmatrix} \tag{80}$$

From the linear stability theory, we know that a fixed point (y_s, a_s) is asymptotically stable when the real part of the eigenvalues of $\mathbf{J}$ are less than 0, i.e., $\mathrm{Re}(\lambda_{\mathbf{J}}) < 0$. For a 2D system, this is equivalent to the conditions: $\mathrm{Tr}(\mathbf{J}) < 0$ and $\det(\mathbf{J}) > 0$. The trace of the Jacobian matrix is given by,

$$\begin{aligned} \mathrm{Tr}(\mathbf{J}) &= \frac{w_r - 1 - w_r\sqrt{a_s}}{\tau_y} + \frac{-1 + wy_s^2}{\tau_a} \\ &= -\left(\frac{1 - w_r + w_r\sqrt{a_s}}{\tau_y} + \frac{1 - wy_s^2}{\tau_a}\right) \\ &= -\left(\frac{1 - w_r + w_r\sqrt{a_s}}{\tau_y} + \frac{b_0^2\sigma^2}{a_s\tau_a}\right) \end{aligned} \tag{81}$$

The determinant of the Jacobian matrix is given by,

$$\begin{aligned} \det(\mathbf{J}) &= \left(\frac{w_r - 1 - w_r\sqrt{a_s}}{\tau_y}\right)\left(\frac{-1 + wy_s^2}{\tau_a}\right) + \left(\frac{w_r y_s}{2\sqrt{a_s}\tau_y}\right)\left(\frac{2wa_s y_s}{\tau_a}\right) \\ &= \frac{(1 - w_r)(1 - wy_s^2)}{\tau_a\tau_y} + \frac{w_r\sqrt{a_s}}{\tau_a\tau_y} \\ &= \frac{1}{\tau_y\tau_a}\left(\frac{(1 - w_r)b_0^2\sigma^2}{a_s} + w_r\sqrt{a_s}\right) \end{aligned} \tag{82}$$

Now we consider the different conditions on w_r and the input drive z for stability and state the various cases as theorems along with their proofs,

E.1 Contractive constraint on recurrence ($0 < w_r \leq 1$)

- $z > 0$: *There exists a unique fixed point with $y_s > 0$ and $a_s > 0$ and it is asymptotically stable.*

  Proof. The existence and uniqueness of the fixed point, along with its stability properties, are established by Lemma E.4. Additionally, the positivity of a_s (i.e., $a_s > 0$) ensures that the expression $1 - w_r + w_r\sqrt{a_s} > 0$. Consequently, given that $z > 0$, it follows from Eq. 77 that $y_s > 0$. $\square$

- $z < 0$: *There exists a unique fixed point with $y_s < 0$ and $a_s > 0$ and it is asymptotically stable.*

  Proof. Since this condition becomes equivalent to $z > 0$, up to a sign change in y (Eq. 74), it is straightforward to see why this is true. □

Lemma E.4. *Given $0 < w_r \leq 1$ and $z \in \mathbb{R}$, there exists a unique fixed point and it is asymptotically stable.*

Proof. We observe that given $0 < w_r \leq 1$, if there exists a valid fixed point, which satisfies $a_s > 0$, $\mathrm{Tr}(\mathbf{J})$ in Eq. 81 is less than 0 (since $1 - w_r + w_r\sqrt{a_s} > 0$ for any $\sqrt{a_s} > 0$), and $\det(\mathbf{J})$ in Eq. 82 is greater than 0 for all combinations of parameters and $z \in \mathbb{R}$. Therefore, we need to find the constraints on the parameters that allow for at least one fixed point. The fixed point, $m = \sqrt{a_s}$, satisfies the quartic polynomial in Eq 79. Due to Theorem E.1, for a valid fixed point, we need to find positive real roots that satisfy $m > 0$.

The sequence of signs of the coefficients of the polynomial when $0 \leq w_r < 1$ is given by $(+, +, \pm, -, -)$. We use *Descartes' Rule of Signs*, which states the following: The number of positive real roots of a polynomial $p(x)$ is either equal to the number of sign changes (omitting the zero coefficients) between consecutive non-zero coefficients of $p(x)$, or it is less than this by a multiple of 2. Since there is exactly one sign change from left to right in the sequence, regardless of the sign of the coefficient of m^2, we know that the equation above has exactly one real positive root for m. This further implies that a_s has exactly one positive root and the corresponding fixed point (y_s, a_s), is locally dynamically stable for all of the combinations of parameters and $z \in \mathbb{R}$.

Note that the result also holds for $w_r = 1$. There is exactly one positive fixed point (y_s, a_s) and it is given by, i.e., $y_s = bz/\sqrt{b_0^2\sigma^2 + wb^2z^2}$ and $a_s = b_0^2\sigma^2 + wb^2z^2$. □

E.2 Expansive constraint on recurrence ($w_r > 1$)

Now we consider the case when $w_r > 1$. We first find an alternative form of the determinant of the Jacobian (Eq.82) that is more amenable to the case, $w_r > 1$,

$$\begin{aligned}\det(\mathbf{J}) &= \frac{1}{\tau_y\tau_a}\left((1 - w_r)(1 - wy_s^2) + w_r\sqrt{a_s}\right)\\ &= \frac{1}{\tau_y\tau_a}\left(1 - w_r + w_r\sqrt{a_s} + (w_r - 1)wy_s^2\right)\end{aligned} \tag{83}$$

Rewriting the trace,

$$\mathrm{Tr}(\mathbf{J}) = -\left(\frac{1 - w_r + w_r\sqrt{a_s}}{\tau_y} + \frac{b_0^2\sigma^2}{a_s\tau_a}\right) \tag{84}$$

The properties of stability can be summarized in the following two cases,

- $z > 0$: *There exists exactly one fixed point with $y_s > 0$ and $a_s > 0$ and it is asymptotically stable. Further, if $b_0\sigma > 1 - 1/w_r$, there exist no additional fixed points. But if $b_0\sigma < 1 - 1/w_r$, then there exist either two or no fixed points with $y_s < 0$ and $a_s > 0$ and they may or may not be stable.*

  Proof. Since the fixed point satisfies Eq. 77, there are two possibilities, either $1 - w_r + w_r\sqrt{a_s} > 0$ or $1 - w_r + w_r\sqrt{a_s} < 0$. We consider them separately,

  - $1 - w_r + w_r\sqrt{a_s} > 0$: If $z > 0$, we have $y_s > 0$ because y_s satisfies Eq. 77. Further due to Lemma E.5, this fixed point is unique and asymptotically stable for all combinations of parameters.
  - $1 - w_r + w_r\sqrt{a_s} < 0$: If $z > 0$, we have $y_s < 0$ because y_s satisfies Eq. 77. Further, if we're given $b_0\sigma > 1 - 1/w_r$, no root exists. We prove this by contradiction, assuming a root exists that satisfies $\sqrt{a_s} < 1 - 1/w_r$, since $b_0\sigma > 1 - 1/w_r$, this root satisfies $0 < \sqrt{a_s} < b_0\sigma$, but due to Lemma E.3 no such root exists, therefore we have a contradiction.
    Now if we have $b_0\sigma < 1 - 1/w_r$, the root for $m = \sqrt{a_s}$ must satisfy $b_0\sigma < m < 1 - 1/w_r$. Since either one or three roots satisfy $m > 0$ (equivalently $m > b_0\sigma$) in

Eq. 79 and exactly one root satisfies $m > 1 - 1/w_r$ (Lemma E.5), we conclude that the number of roots for m in the interval $(b_0\sigma, 1 - 1/w_r)$ are either two or none.

□

- $z < 0$: *There exists exactly one fixed point with* $y_s < 0$ *and* $a_s > 0$ *and it is asymptotically stable. Further, if* $b_0\sigma > 1 - 1/w_r$*, there exist no additional fixed points. But if* $b_0\sigma < 1 - 1/w_r$*, then there exist either two or no fixed points with* $y_s > 0$ *and* $a_s > 0$ *and they may or may not be stable.*

  Proof. Since this condition becomes equivalent to $z > 0$, up to a sign change in y (Eq. 74), it is straightforward to see why this is true. □

Lemma E.5. *Given* $w_r > 1$*, there exists a unique fixed point,* (y_s, a_s)*, that satisfies* $1-w_r+w_r\sqrt{a_s} > 0$ *and it is asymptotically stable.*

Proof. The fixed point, $m = \sqrt{a_s}$, satisfies the quartic polynomial in Eq 79. Due to Theorem E.1, for a valid fixed point, we need to find positive real roots that satisfy $m > 0$.

The sequence of signs of the coefficients of the polynomial is given by $(+, -, \pm, +, -)$. Regardless of the sign of the coefficient of m^2, using *Descartes' Rule of Signs*, we find that since there are 3 sign changes, there are either 1 or 3 positive real roots for m. Since, we're given $w_r > 1$ and $1 - w_r + w_r\sqrt{a_s} > 0$, these roots are valid only if they satisfy $1 - w_r + w_r m > 0$, or, $m > 1 - 1/w_r$. We make a transformation $m \to \hat{m} + (1 - 1/w_r)$ which gives us,

$$\begin{aligned} {w_r}^2\hat{m}^4 - 2(1 - w_r)w_r\hat{m}^3 + \left((1 - w_r)^2 - wb^2z^2 - b_0^2\sigma^2{w_r}^2\right)\hat{m}^2 \\ - 2wb^2z^2(1 - 1/w_r)\hat{m} - wb^2z^2(1 - 1/w_r)^2 = 0 \end{aligned} \tag{85}$$

The number of valid roots is given by the number of positive real roots of this polynomial. The signs of the coefficients are given by, $(+, +, \pm, -, -)$. There is exactly one sign change, hence there is a unique fixed point that satisfies $1 - w_r + w_r\sqrt{a_s} > 0$.

Now we prove that this fixed point is asymptotically stable. It can be easily seen that $\mathrm{Tr}(\mathbf{J}) < 0$ in Eq. 84 and $\det(\mathbf{J}) > 0$ in Eq. 83 when $1 - w_r + w_r\sqrt{a_s} > 0$ and $w_r > 1$. Therefore, this unique fixed point is asymptotically stable. □

F Analysis of the Rectified model

Here, we present the stability results for the model with only the positive one of the complementary receptive fields present. The model is given by the following dynamical equations,

$$\begin{aligned} \boldsymbol{\tau}_y \odot \dot{\mathbf{y}} &= -\mathbf{y} + \mathbf{b} \odot \mathbf{z} + \left(\mathbf{1} - \mathbf{a}^+\right) \odot \mathbf{W}_r \left(\sqrt{\mathbf{y}^+}\right) \\ \boldsymbol{\tau}_a \odot \dot{\mathbf{a}} &= -\mathbf{a} + \mathbf{b}_0^2 \odot \boldsymbol{\sigma}^2 + \mathbf{W}\left(\mathbf{y}^+ \odot \mathbf{a}^{+2}\right) \end{aligned} \tag{86}$$

We will follow the same procedure for stability analysis as we did for the main model and state the key steps in the various derivations for stability.

F.1 Stability of high dimensional system

We analyze the stability of the system when $\mathbf{W}_r = \mathbf{I}$. Upon substituting $\mathbf{y}^+ \to \lfloor \mathbf{y} \rfloor^2$ and $\mathbf{a}^+ \to \sqrt{\lfloor \mathbf{a} \rfloor}$, we get the following dynamical system,

$$\begin{aligned} \boldsymbol{\tau}_y \odot \dot{\mathbf{y}} &= -\mathbf{y} + \mathbf{b} \odot \mathbf{z} + \left(\mathbf{1} - \sqrt{\lfloor \mathbf{a} \rfloor}\right) \odot \lfloor \mathbf{y} \rfloor \\ \boldsymbol{\tau}_a \odot \dot{\mathbf{a}} &= -\mathbf{a} + \mathbf{b}_0^2 \odot \boldsymbol{\sigma}^2 + \mathbf{W}\left(\lfloor \mathbf{y} \rfloor^2 \odot \lfloor \mathbf{a} \rfloor\right) \end{aligned} \tag{87}$$

The fixed point is given by,

$$\begin{aligned} \mathbf{y}_s &= \frac{\lfloor \mathbf{b} \odot \mathbf{z} \rfloor}{\sqrt{\mathbf{b}_0^2 \odot \boldsymbol{\sigma}^2 + \mathbf{W}\lfloor \mathbf{b} \odot \mathbf{z} \rfloor^2}} - \lfloor -\mathbf{b} \odot \mathbf{z} \rfloor \\ \mathbf{a}_s &= \mathbf{b}_0^2 \odot \boldsymbol{\sigma}^2 + \mathbf{W}\lfloor \mathbf{b} \odot \mathbf{z} \rfloor^2 \end{aligned} \tag{88}$$

Also, $\lfloor \mathbf{y}_s \rfloor$ is given by,

$$\lfloor \mathbf{y}_s \rfloor = \frac{\lfloor \mathbf{b} \odot \mathbf{z} \rfloor}{\sqrt{\mathbf{b}_0^2 \odot \boldsymbol{\sigma}^2 + \mathbf{W} \lfloor \mathbf{b} \odot \mathbf{z} \rfloor^2}} \tag{89}$$

The Jacobian matrix, $\mathbf{J}$, about this fixed point is given by,

$$\mathbf{J} = \begin{bmatrix} -\mathbf{D}\left(\frac{\sqrt{\mathbf{a}_s} \odot \mathbf{1}_{\mathbf{z} \geq 0} + \mathbf{1}_{\mathbf{z}<0}}{\boldsymbol{\tau}_y}\right) & -\mathbf{D}\left(\frac{\lfloor \mathbf{y}_s \rfloor}{\mathbf{2} \odot \sqrt{\mathbf{a}_s} \odot \boldsymbol{\tau}_y}\right) \\ \mathbf{D}\left(\frac{\mathbf{2}}{\boldsymbol{\tau}_a}\right) \mathbf{W} \, \mathbf{D}\left(\mathbf{a}_s \odot \lfloor \mathbf{y}_s \rfloor\right) & \mathbf{D}\left(\frac{\mathbf{1}}{\boldsymbol{\tau}_a}\right)\left(-\mathbf{I} + \mathbf{W} \, \mathbf{D}\left(\lfloor \mathbf{y}_s \rfloor^2\right)\right) \end{bmatrix} \tag{90}$$

Here $\mathbf{1}_{\mathbf{z} \geq 0}$ is an indicator function that returns a vector the size of $\mathbf{z}$ with 1 at locations where $z_i \geq 0$ and 0 elsewhere. Similarly, $\mathbf{1}_{\mathbf{z}<0}$ returns a vector the size of $\mathbf{z}$ with 1 at locations where $z_i < 0$ and 0 elsewhere. Further $\mathbf{J} - \lambda \mathbf{I}$ is given by,

$$\begin{aligned} \mathbf{J} &= \begin{bmatrix} \mathbf{A}_{11} & \mathbf{A}_{12} \\ \mathbf{A}_{21} & \mathbf{A}_{22} \end{bmatrix} \\ &= \begin{bmatrix} -\mathbf{D}\left(\frac{\sqrt{\mathbf{a}_s} \odot \mathbf{1}_{\mathbf{z} \geq 0} + \mathbf{1}_{\mathbf{z}<0}}{\boldsymbol{\tau}_y}\right) - \lambda \mathbf{I} & -\mathbf{D}\left(\frac{\lfloor \mathbf{y}_s \rfloor}{\mathbf{2} \odot \sqrt{\mathbf{a}_s} \odot \boldsymbol{\tau}_y}\right) \\ \mathbf{D}\left(\frac{\mathbf{2}}{\boldsymbol{\tau}_a}\right) \mathbf{W} \, \mathbf{D}\left(\mathbf{a}_s \odot \lfloor \mathbf{y}_s \rfloor\right) & \mathbf{D}\left(\frac{\mathbf{1}}{\boldsymbol{\tau}_a}\right)\left(-\mathbf{I} + \mathbf{W} \, \mathbf{D}\left(\lfloor \mathbf{y}_s \rfloor^2\right)\right) - \lambda \mathbf{I} \end{bmatrix} \end{aligned} \tag{91}$$

Since $\mathbf{A}_{11}$ and $\mathbf{A}_{12}$ commute, we can write, $\det(\mathbf{J} - \lambda \mathbf{I}) = \det(\mathbf{A}_{22}\mathbf{A}_{11} - \mathbf{A}_{21}\mathbf{A}_{12})$. Upon simplification, we get,

$$\begin{aligned} \det(\mathbf{J} - \lambda \mathbf{I}) = \det\Bigg(\lambda^2 \mathbf{I} + \lambda \Bigg[\mathbf{D}\left(\frac{\mathbf{1}}{\boldsymbol{\tau}_a}\right) + \mathbf{D}\left(\frac{\sqrt{\mathbf{a}_s} \odot \mathbf{1}_{\mathbf{z} \geq 0} + \mathbf{1}_{\mathbf{z}<0}}{\boldsymbol{\tau}_y}\right) \\ - \mathbf{D}\left(\frac{\mathbf{1}}{\boldsymbol{\tau}_a}\right) \mathbf{W} \mathbf{D}\left(\lfloor \mathbf{y}_s \rfloor^2\right)\Bigg] + \mathbf{D}\left(\frac{\sqrt{\mathbf{a}_s} \odot \mathbf{1}_{\mathbf{z} \geq 0} + \mathbf{1}_{\mathbf{z}<0}}{\boldsymbol{\tau}_y \odot \boldsymbol{\tau}_a}\right)\Bigg) \end{aligned} \tag{92}$$

Note that, here we used the fact that $\mathbf{y}_s$ has the same sign as $\mathbf{z}$, as seen from Eq. 88. The dynamical system is stable if all eigenvalues of this characteristic polynomial have negative real parts. Just like the main model, we map this to a quadratic eigenvalue problem of the form $\mathcal{L}(\lambda) = \det(\lambda^2 \mathbf{I} + \lambda \mathbf{B} + \mathbf{K}) = 0$. Now, we see if the conditions of Theorem 4.1 are met. The stiffness matrix is given by,

$$\mathbf{K} = \mathbf{D}\left(\frac{\sqrt{\mathbf{a}_s} \odot \mathbf{1}_{\mathbf{z} \geq 0} + \mathbf{1}_{\mathbf{z}<0}}{\boldsymbol{\tau}_y \odot \boldsymbol{\tau}_a}\right) = \mathbf{D}\left(\frac{\sqrt{\mathbf{b}_0^2 \odot \boldsymbol{\sigma}^2 + \mathbf{W} \lfloor \mathbf{b} \odot \mathbf{z} \rfloor^2} \odot \mathbf{1}_{\mathbf{z} \geq 0} + \mathbf{1}_{\mathbf{z}<0}}{\boldsymbol{\tau}_y \odot \boldsymbol{\tau}_a}\right) \tag{93}$$

Clearly, $\mathbf{K}$ is a positive diagonal matrix for all choices of parameters and input. Now we check if the $\mathbf{B}$ is a Lyapunov diagonally stable matrix, which is implied when $\mathbf{B}$ admits a *regular convergent splitting*, i.e., it has a representation of the form $\mathbf{B} = \mathbf{M} - \mathbf{N}$, where $\mathbf{M}^{-1}$ and $\mathbf{N}$ have all nonnegative entries and $\mathbf{M}^{-1}\mathbf{N}$ has a spectral radius smaller than 1.

$$\mathbf{B} = \underbrace{\mathbf{D}\left(\frac{\mathbf{1}}{\boldsymbol{\tau}_a}\right) + \mathbf{D}\left(\frac{\sqrt{\mathbf{a}_s} \odot \mathbf{1}_{\mathbf{z} \geq 0} + \mathbf{1}_{\mathbf{z}<0}}{\boldsymbol{\tau}_y}\right)}_{\mathbf{M}} - \underbrace{\mathbf{D}\left(\frac{\mathbf{1}}{\boldsymbol{\tau}_a}\right) \mathbf{W} \mathbf{D}\left(\lfloor \mathbf{y}_s \rfloor^2\right)}_{\mathbf{N}} \tag{94}$$

Since $\mathbf{M}$ is a positive diagonal matrix, all the entries of $\mathbf{M}^{-1}$ are nonnegative. Also, since all the matrices involved in the definition of $\mathbf{N}$ are nonnegative, therefore their product, $\mathbf{N}$, is nonnegative. We're only left to prove that $\mathbf{M}^{-1}\mathbf{N} = \mathbf{S}$ has a spectral radius smaller than 1. Consider the matrix $\mathbf{S}$,

$$\mathbf{S} = \mathbf{D}\left(\frac{\mathbf{1}}{\mathbf{1} + (\boldsymbol{\tau}_a / \boldsymbol{\tau}_y) \odot \left(\sqrt{\mathbf{a}_s} \odot \mathbf{1}_{\mathbf{z} \geq 0} + \mathbf{1}_{\mathbf{z}<0}\right)}\right) \mathbf{W} \mathbf{D}\left(\frac{\lfloor \mathbf{b} \odot \mathbf{z} \rfloor^2}{\mathbf{b}_0^2 \odot \boldsymbol{\sigma}^2 + \mathbf{W} \lfloor \mathbf{b} \odot \mathbf{z} \rfloor^2}\right) \tag{95}$$

Theorem D puts a bound of 1 on the spectral radius of this matrix. Define $\mathbf{t} \rightarrow \mathbf{1} / \left(\mathbf{1} + (\boldsymbol{\tau}_a / \boldsymbol{\tau}_y) \odot \left(\sqrt{\mathbf{a}_s} \odot \mathbf{1}_{\mathbf{z} \geq 0} + \mathbf{1}_{\mathbf{z}<0}\right)\right)$, $\mathbf{u} \rightarrow \lfloor \mathbf{b} \odot \mathbf{z} \rfloor^2$ and $\mathbf{v} \rightarrow \mathbf{b}_0^2 \odot \boldsymbol{\sigma}^2$, we notice that they follow the constraints of the theorem, therefore, $\mathbf{S}$ is convergent. This implies that $\mathbf{B}$ has a *convergent regular splitting*, therefore, the linearized dynamical system is unconditionally globally asymptotically stable (and nonlinear dynamical system is locally asymptotically stable) across all the values of parameters and inputs.

F.2 Analytical eigenvalue for fully normalized circuit

Following a procedure similar to that in Appendix C, when all of the normalization weights in the system are equal, to value α, and the various parameters are scalars, i.e., $\boldsymbol{\tau}_y = \tau_y \mathbf{1}$, $\boldsymbol{\tau}_a = \tau_a \mathbf{1}$, $\mathbf{b}_0 = b_0 \mathbf{1}$ and $\boldsymbol{\sigma} = \sigma \mathbf{1}$, we can write the analytical expressions for eigenvalues. The characteristic polynomial is given by,

$$\det(\mathbf{J} - \lambda \mathbf{I}) = \left(1 - \frac{\lambda \alpha}{\tau_a} \mathbf{v}^\top \mathbf{D} \left(\frac{\mathbf{1}}{\delta_1 \mathbf{1}_{\mathbf{z} \geq 0} + \delta_2 \mathbf{1}_{\mathbf{z} < 0}} \right) \mathbf{u} \right) \delta_1^{n_1} \delta_2^{n_2} \tag{96}$$

where, $\mathbf{u} = [1, 1, ..., 1]^\top$, $\mathbf{v} = \lfloor \mathbf{b} \odot \mathbf{z} \rfloor^2 / \left(\sigma^2 b_0^2 \mathbf{1} + \mathbf{W} \lfloor \mathbf{b} \odot \mathbf{z} \rfloor^2 \right)$; n_1 and n_2 are the number of nonnegative and negative values, respectively, in the input drive $\mathbf{z}$; δ_1 and δ_2 are given by,

$$\begin{aligned} \delta_1 &= \lambda^2 + \lambda \left(\frac{1}{\tau_a} + \frac{\sqrt{\sigma^2 b_0^2 + \alpha ||\lfloor \mathbf{b} \odot \mathbf{z} \rfloor||^2}}{\tau_y} \right) + \frac{\sqrt{\sigma^2 b_0^2 + \alpha ||\lfloor \mathbf{b} \odot \mathbf{z} \rfloor||^2}}{\tau_y \tau_a} \\ \delta_2 &= \lambda^2 + \lambda \left(\frac{1}{\tau_a} + \frac{1}{\tau_y} \right) + \frac{1}{\tau_y \tau_a} \end{aligned} \tag{97}$$

Simplification of Eq. 96 gives us,

$$\det(\mathbf{J} - \lambda \mathbf{I}) = \left(\delta_1 - \frac{\lambda}{\tau_a} \frac{\alpha ||\lfloor \mathbf{b} \odot \mathbf{z} \rfloor||^2}{\sigma^2 b_0^2 + \alpha ||\lfloor \mathbf{b} \odot \mathbf{z} \rfloor||^2} \right) \delta_1^{n_1 - 1} \delta_2^{n_2} \tag{98}$$

Since the characteristic polynomial is a product of quadratic polynomials, we can solve them analytically. The strictly negative eigenvalues are given by,

$$\lambda = -\frac{1}{\tau_a}; \quad \lambda = -\frac{1}{\tau_y} \quad \& \quad \lambda = -\frac{\sqrt{\sigma^2 b_0^2 + \alpha ||\lfloor \mathbf{b} \odot \mathbf{z} \rfloor||^2}}{\tau_y} \tag{99}$$

The potentially complex eigenvalues are given by the solution to the following quadratic equation,

$$\lambda^2 + \lambda \left(\frac{1}{\tau_a} \left(1 - \frac{\alpha ||\lfloor \mathbf{b} \odot \mathbf{z} \rfloor||^2}{\sigma^2 b_0^2 + \alpha ||\lfloor \mathbf{b} \odot \mathbf{z} \rfloor||^2} \right) + \frac{\sqrt{\sigma^2 b_0^2 + \alpha ||\lfloor \mathbf{b} \odot \mathbf{z} \rfloor||^2}}{\tau_y} \right) + \frac{\sqrt{\sigma^2 b_0^2 + \alpha ||\lfloor \mathbf{b} \odot \mathbf{z} \rfloor||^2}}{\tau_y \tau_a} = 0 \tag{100}$$

F.3 Linear stability analysis of the two-dimensional model

The dynamical system to consider is,

$$\begin{aligned} \tau_y \dot{y} &= -y + bz + \left(1 - \sqrt{\lfloor a \rfloor}\right) w_r \lfloor y \rfloor \\ \tau_a \dot{a} &= -a + b_0^2 \sigma^2 + w \lfloor y \rfloor^2 \lfloor a \rfloor \end{aligned} \tag{101}$$

Since a valid fixed point must have $a_s > 0$, the fixed point (y_s, a_s) satisfies,

$$\left(1 - w_r + w_r \sqrt{a_s}\right) \lfloor y_s \rfloor - \lfloor -y_s \rfloor = bz \tag{102}$$

$$\left(1 - w \lfloor y_s \rfloor^2\right) a_s = b_0^2 \sigma^2 \tag{103}$$

We divide this into two cases,

- $y_s > 0$: The fixed point is given by the equations,

$$\left(1 - w_r + w_r \sqrt{a_s}\right) y_s = bz \tag{104}$$

$$\left(1 - w y_s^2\right) a_s = b_0^2 \sigma^2 \tag{105}$$

A fixed point, (y_s, a_s), is valid only if it satisfies $y_s \in \mathbb{R}_*^+$ and $a_s \in \mathbb{R}_*^+$. The Jacobian matrix is given by,

$$\mathbf{J} = \begin{bmatrix} \frac{w_r - 1 - w_r \sqrt{a_s}}{\tau_y} & -\frac{w_r y_s}{2\sqrt{a_s} \tau_y} \\ \frac{2 w a_s y_s}{\tau_a} & \frac{-1 + w y_s^2}{\tau_a} \end{bmatrix} \tag{106}$$

Note that this is equivalent to the main model, with the additional constraint of $y_s > 0$, whose stability analysis is presented in Appendix E.

- $y_s < 0$: Eq. 102 & 103 yield us a unique fixed point $y_s = bz$ and $a_s = b_0^2\sigma^2$. Since $y_s < 0$ and $y_s = bz$, this is only possible when $z < 0$. The Jacobian matrix about (y_s, a_s) is given by,

$$\mathbf{J} = \begin{bmatrix} -\frac{1}{\tau_y} & 0 \\ 0 & -\frac{1}{\tau_a} \end{bmatrix} \tag{107}$$

  Since the eigenvalues of $\mathbf{J}$ are $\lambda_{\mathbf{J}} = -1/\tau_y, -1/\tau_a$ are real and both negative, this fixed point is always stable.

Combining the cases above and results already established in Appendix E, we characterize the stability of the system as follows,

F.3.1 Contractive constraint on recurrence ($0 < w_r \leq 1$)

- $z > 0$: *There exists a unique fixed point with $y_s > 0$ and $a_s > 0$ and it is asymptotically stable.*
- $z < 0$: *There exists a unique fixed point with $y_s < 0$ and $a_s > 0$, given by, $(bz, b_0^2\sigma^2)$, and it is asymptotically stable.*

F.3.2 Expansive constraint on recurrence ($w_r > 1$)

- $z > 0$: *There exists a unique fixed point with $y_s > 0$ and $a_s > 0$ and it is asymptotically stable.*
- $z < 0$: *There exists exactly one fixed point with $y_s < 0$ and $a_s > 0$ given by, $(bz, b_0^2\sigma^2)$, and it is asymptotically stable. Further, if $b_0\sigma > 1 - 1/w_r$, there exist no additional fixed points. But if $b_0\sigma < 1 - 1/w_r$, then there exist either two or no fixed points with $y_s > 0$ and $a_s > 0$ and they may or may not be stable.*

G Iterative algorithm

In this section, we present an iterative approach to finding the fixed point for ORGaNICs with an arbitrary recurrent weight matrix. We show that this algorithm converges in a few steps (2-10) with great accuracy. We consider the system given by Eq. 14. We restate the equations here for completeness,

$$\begin{aligned} \boldsymbol{\tau}_y \odot \dot{\mathbf{y}} &= -\mathbf{y} + \mathbf{b} \odot \mathbf{z} + \left(\mathbf{1} - \sqrt{\lfloor \mathbf{a} \rfloor}\right) \odot \mathbf{W}_r \mathbf{y} \\ \boldsymbol{\tau}_a \odot \dot{\mathbf{a}} &= -\mathbf{a} + \mathbf{b}_0^2 \odot \boldsymbol{\sigma}^2 + \mathbf{W} \left(\mathbf{y}^2 \odot \lfloor \mathbf{a} \rfloor\right) \end{aligned} \tag{108}$$

The fixed point of this system ($\mathbf{y}_s$ and $\mathbf{a}_s$) is given by the solution of the following simultaneous equations,

$$\mathbf{y}_s = \mathbf{b} \odot \mathbf{z} + (\mathbf{1} - \sqrt{\mathbf{a}_s}) \odot (\mathbf{W}_r \mathbf{y}_s) \tag{109}$$

$$\mathbf{a}_s = \mathbf{b}_0^2 \odot \boldsymbol{\sigma}^2 + \mathbf{W} \left(\mathbf{y}_s^2 \odot \mathbf{a}_s\right) \tag{110}$$

These equations do not admit a closed-form analytical solution when $\mathbf{W}_r \neq \mathbf{I}$. The fast convergence is owed to the fact that we have a good initial approximation of the solution. We first find a good approximation for the initialization of $\mathbf{y}_s$ and $\mathbf{a}_s$ for the iterative algorithm. The equation for $\mathbf{y}_s$ can be written in terms of $\mathbf{a}_s$ as,

$$\mathbf{y}_s = \left(\mathbf{I} + \left(\mathbf{D}\left(\sqrt{\mathbf{a}_s}\right) - \mathbf{I}\right) \mathbf{W}_r\right)^{-1} (\mathbf{b} \odot \mathbf{z}) \tag{111}$$

Now applying the Woodbury matrix identity which states that,

$$\left(\mathbf{A} + \mathbf{UCV}\right)^{-1} = \mathbf{A}^{-1} - \mathbf{A}^{-1}\mathbf{U}\left(\mathbf{C}^{-1} + \mathbf{VA}^{-1}\mathbf{U}\right)^{-1} \mathbf{VA}^{-1} \tag{112}$$

to the inverse in Eq. 111 with $\mathbf{A} = \mathbf{I}$, $\mathbf{U} = \mathbf{I}$, $\mathbf{C} = \mathbf{D}\left(\sqrt{\mathbf{a}_s}\right) - \mathbf{I}$ and $\mathbf{V} = \mathbf{W}_r$, we get,

$$\mathbf{y}_s = \left(\mathbf{I} - \left(\left(\mathbf{D}\left(\sqrt{\mathbf{a}_s}\right) - \mathbf{I}\right)^{-1} + \mathbf{W}_r\right)^{-1} \mathbf{W}_r\right) (\mathbf{b} \odot \mathbf{z}) \tag{113}$$

We approximate the above equation by assuming that $\mathbf{W}_r$ is a symmetric matrix with the eigendecomposition given by $\mathbf{Q\Lambda Q}^\top$, with $\mathbf{Q}^\top\mathbf{Q} = \mathbf{QQ}^\top = \mathbf{I}$ and $\mathbf{\Lambda} = \mathbf{D}(\boldsymbol{\lambda})$ is a diagonal matrix with the eigenvalues as its diagonal entries. This gives us the following approximation,

$$
\begin{aligned}
\mathbf{y}_s &\approx \left(\mathbf{I} - \left(\mathbf{Q}\left(\left(\mathbf{D}\left(\sqrt{\mathbf{a}_s}\right) - \mathbf{I}\right)^{-1} + \mathbf{\Lambda}\right)\mathbf{Q}^\top\right)^{-1}\mathbf{Q\Lambda Q}^\top\right)(\mathbf{b}\odot\mathbf{z}) \\
&= \left(\mathbf{I} - \mathbf{QD}\left(\boldsymbol{\lambda} + \frac{\mathbf{1}}{\sqrt{\mathbf{a}_s} - \mathbf{1}}\right)^{-1}\mathbf{Q}^\top\mathbf{Q\Lambda Q}^\top\right)(\mathbf{b}\odot\mathbf{z}) \\
&= \left(\mathbf{I} - \mathbf{QD}\left(\frac{\boldsymbol{\lambda} * \sqrt{\mathbf{a}_s} - \boldsymbol{\lambda}}{\mathbf{1} - \boldsymbol{\lambda} + \boldsymbol{\lambda} * \sqrt{\mathbf{a}_s}}\right)\mathbf{Q}^\top\right)(\mathbf{b}\odot\mathbf{z}) \\
&= \left(\mathbf{I} - \mathbf{Q}\left(\mathbf{I} - \mathbf{D}\left(\frac{\mathbf{1}}{\mathbf{1} - \boldsymbol{\lambda} + \boldsymbol{\lambda} * \sqrt{\mathbf{a}_s}}\right)\right)\mathbf{Q}^\top\right)(\mathbf{b}\odot\mathbf{z}) \\
&= \mathbf{QD}\left(\frac{\mathbf{1}}{\mathbf{1} - \boldsymbol{\lambda} + \boldsymbol{\lambda} * \sqrt{\mathbf{a}_s}}\right)\mathbf{Q}^\top(\mathbf{b}\odot\mathbf{z}) \\
&= \mathbf{QD}\left(\frac{\mathbf{1}}{\boldsymbol{\lambda} - \boldsymbol{\lambda}^2 + \boldsymbol{\lambda}^2 * \sqrt{\mathbf{a}_s}}\right)\mathbf{\Lambda Q}^\top(\mathbf{b}\odot\mathbf{z})
\end{aligned}
\tag{114}
$$

We approximate the eigenvalues, $\boldsymbol{\lambda}$, by the maximum eigenvalue of the $\mathbf{W}_r$ and assume the entries of $\sqrt{\mathbf{a}_s}$ are identical. This gives us the following initial guess for $\mathbf{y}_s$.

$$
\begin{aligned}
\mathbf{y}_s^0 &= \mathbf{D}\left(\frac{\mathbf{1}}{\lambda_m - \lambda_m^2 + \lambda_m^2\sqrt{\mathbf{a}_s^0}}\right)\mathbf{Q\Lambda Q}^\top(\mathbf{b}\odot\mathbf{z}) \\
&= \frac{\mathbf{W}_r(\mathbf{b}\odot\mathbf{z})}{\lambda_m - \lambda_m^2 + \lambda_m^2\sqrt{\mathbf{a}_s^0}}
\end{aligned}
\tag{115}
$$

For the initial guess of $\mathbf{a}_s^0$, we use Eq. 110 and plug in the following on the RHS $\mathbf{a}_s \to \mathbf{b}_0^2 \odot \boldsymbol{\sigma}^2$ and the corresponding $\mathbf{y}_s$ found by using Eq. 115. This gives us the following,

$$
\mathbf{a}_s^0 = \boldsymbol{\sigma}^2 \odot \mathbf{b}_0^2 + \mathbf{W}\left(\left(\frac{\mathbf{W}_r(\mathbf{b}\odot\mathbf{z})}{\lambda_m - \lambda_m^2 + \lambda_m^2\sqrt{\mathbf{b}_0^2\odot\boldsymbol{\sigma}^2}}\right)^2 \odot \left(\mathbf{b}_0^2 \odot \boldsymbol{\sigma}^2\right)\right)
\tag{116}
$$

Next we update the $\mathbf{y}_s$ and $\mathbf{a}_s$ by performing the following iterations derived using Eq. 109 & 110. For instance, $(\mathbf{y}_s^1, \mathbf{a}_s^1)$ are given by,

$$
\begin{aligned}
\mathbf{y}_s^1 &= \left(\mathbf{I} - \mathbf{W}_r + \mathbf{D}\left(\sqrt{\mathbf{a}_s^0}\right)\mathbf{W}_r\right)^{-1}(\mathbf{b}\odot\mathbf{z}) \\
\mathbf{a}_s^1 &= \mathbf{b}_0^2 \odot \boldsymbol{\sigma}^2 + \mathbf{W}\left(\left(\mathbf{y}_s^1\right)^2 * \mathbf{a}_s^0\right)
\end{aligned}
\tag{117}
$$

This procedure is summarized in Algorithm 2. Even though we had assumed that $\mathbf{W}_r$ should be symmetric, in practice we find that this algorithm leads to fast convergence even for non-symmetric matrices. We also find that this iteration scheme works only for recurrent weight matrices with a maximum singular value of 1.

H Energy function of ORGaNICs

Here, we find the energy (Lyapunov) function that is minimized by the dynamics of the ORGaNICs circuit in the vicinity of the normalization fixed point. We consider the dynamical system with $\mathbf{W}_r = \mathbf{I}$, which is given by Eq. 3. Upon linearizing about the fixed point we get the following linear system,

$$
\begin{bmatrix}\dot{\mathbf{y}} \\ \dot{\mathbf{a}}\end{bmatrix} = \begin{bmatrix} -\mathbf{D}\left(\frac{\sqrt{\mathbf{a}_s}}{\boldsymbol{\tau}_y}\right) & -\mathbf{D}\left(\frac{\mathbf{y}_s}{\mathbf{2}\odot\sqrt{\mathbf{a}_s}\odot\boldsymbol{\tau}_y}\right) \\ \mathbf{D}\left(\frac{\mathbf{2}}{\boldsymbol{\tau}_a}\right)\mathbf{W}\,\mathbf{D}\left(\mathbf{a}_s\odot\mathbf{y}_s\right) & \mathbf{D}\left(\frac{\mathbf{1}}{\boldsymbol{\tau}_a}\right)\left(-\mathbf{I} + \mathbf{W}\,\mathbf{D}\left(\mathbf{y}_s^2\right)\right)\end{bmatrix}\begin{bmatrix}\mathbf{y} - \mathbf{y}_s \\ \mathbf{a} - \mathbf{a}_s\end{bmatrix}
\tag{118}
$$

Algorithm 2 Iterative scheme for finding the fixed point

Input: ORGaNICs parameters and input $(\mathbf{z})$, Tolerance ϵ, maximum iterations N
Output: Approximation to the fixed point $(\mathbf{y}_s, \mathbf{a}_s)$
$\mathbf{a} \leftarrow \boldsymbol{\sigma}^2 \odot \mathbf{b}_0^2 + \mathbf{W}\left(\left(\frac{\mathbf{W}_r(\mathbf{b}\odot\mathbf{z})}{\lambda_m - \lambda_m^2 + \lambda_m^2\sqrt{\mathbf{b}_0^2\odot\boldsymbol{\sigma}^2}}\right)^2 \odot \left(\mathbf{b}_0^2 \odot \boldsymbol{\sigma}^2\right)\right)$
$\mathbf{y} \leftarrow \frac{\mathbf{W}_r(\mathbf{b}\odot\mathbf{z})}{\lambda_m - \lambda_m^2 + \lambda_m^2\sqrt{\mathbf{a}}}$
$k \leftarrow 0$
while $\|\mathbf{y} - \mathbf{b}\odot\mathbf{z} - \left(\mathbf{1} - \sqrt{\mathbf{a}}\right) \odot (\mathbf{W}_r\mathbf{y})\| > \epsilon$ and $k < N$ **do**
    $\mathbf{y} \leftarrow \left(\mathbf{I} - \mathbf{W}_r + \mathbf{D}\left(\sqrt{\mathbf{a}}\right)\mathbf{W}_r\right)^{-1}(\mathbf{b}\odot\mathbf{z})$
    $\mathbf{a} \leftarrow \mathbf{b}_0^2 \odot \boldsymbol{\sigma}^2 + \mathbf{W}\left(\mathbf{y}^2 * \mathbf{a}\right)$
    $k \leftarrow k + 1$
end while
return $(\mathbf{y}, \mathbf{a})$

This system is dynamically equivalent to a system of coupled harmonic oscillators with the following equations,

$$\ddot{\mathbf{x}} + \left[\mathbf{D}\left(\frac{\mathbf{1}}{\boldsymbol{\tau}_a}\right) + \mathbf{D}\left(\frac{\sqrt{\mathbf{a}_s}}{\boldsymbol{\tau}_y}\right) - \mathbf{D}\left(\frac{\mathbf{1}}{\boldsymbol{\tau}_a}\right)\mathbf{W}\mathbf{D}\left(\mathbf{y}_s^2\right)\right]\dot{\mathbf{x}} + \mathbf{D}\left(\frac{\sqrt{\mathbf{a}_s}}{\boldsymbol{\tau}_y \odot \boldsymbol{\tau}_a}\right)\mathbf{x} = \mathbf{0} \tag{119}$$

or, we can write the linear system in terms of the position, $\mathbf{x}$, and the velocity, $\mathbf{v}$,

$$\begin{bmatrix}\dot{\mathbf{x}}\\ \dot{\mathbf{v}}\end{bmatrix} = \begin{bmatrix}\mathbf{0} & \mathbf{I}\\ -\mathbf{D}\left(\frac{\sqrt{\mathbf{a}_s}}{\boldsymbol{\tau}_y\odot\boldsymbol{\tau}_a}\right) & -\left[\mathbf{D}\left(\frac{\mathbf{1}}{\boldsymbol{\tau}_a}\right) + \mathbf{D}\left(\frac{\sqrt{\mathbf{a}_s}}{\boldsymbol{\tau}_y}\right) - \mathbf{D}\left(\frac{\mathbf{1}}{\boldsymbol{\tau}_a}\right)\mathbf{W}\mathbf{D}\left(\mathbf{y}_s^2\right)\right]\end{bmatrix}\begin{bmatrix}\mathbf{x}\\ \mathbf{v}\end{bmatrix} \tag{120}$$

Since this system is of the form $\mathbf{I}\ddot{\mathbf{x}} + \mathbf{B}\dot{\mathbf{x}} + \mathbf{K}\mathbf{x} = \mathbf{0}$, Eq. 35, the Lyapunov/energy function of this dynamical system is given by $V(\mathbf{z}) = \mathbf{z}^\top\mathbf{P}\mathbf{z}$, or,

$$V(\mathbf{x}, \mathbf{v}) = \begin{bmatrix}\mathbf{x}^\top & \mathbf{v}^\top\end{bmatrix}\begin{bmatrix}\mathbf{TK} & \epsilon\mathbf{I}\\ \epsilon\mathbf{I} & \mathbf{T}\end{bmatrix}\begin{bmatrix}\mathbf{x}\\ \mathbf{v}\end{bmatrix} = \mathbf{x}^\top(\mathbf{TK})\,\mathbf{x} + \mathbf{v}^\top\mathbf{T}\mathbf{v} + 2\epsilon\,\mathbf{x}^\top\mathbf{v} \tag{121}$$

Here, $\mathbf{T}$ is any positive diagonal matrix such that $\mathbf{TB} + \mathbf{B}^\top\mathbf{T} \succ 0$ and $\mathbf{K}$ is also a positive diagonal matrix given by $\mathbf{D}\left(\sqrt{\mathbf{a}_s}/\left(\boldsymbol{\tau}_y \odot \boldsymbol{\tau}_a\right)\right)$. Now, for a valid Lyapunov function, we can take ϵ to be arbitrarily small Eq. 50. Therefore, the energy minimized by the dynamical system is given by $V(\mathbf{x}, \mathbf{v}) = \mathbf{x}^\top(\mathbf{TK})\,\mathbf{x} + \mathbf{v}^\top\mathbf{T}\mathbf{v}$. This is a high-dimensional version of the energy of a damped harmonic oscillator. For a single oscillator, we have $V(x, v) = t(kx^2 + v^2)$ which is proportional to the total energy (kinetic + potential) of the oscillator.

We now express this energy function in terms of the variables relevant to ORGaNICs, i.e., we find $V(y, a)$. First, we denote the Jacobian matrices in RHS of Eq. 118 & 120 by $\mathbf{A}$ & $\mathbf{B}$, respectively. We note the simple fact that $\mathbf{A}$ & $\mathbf{B}$ are related by a similarity transformation (a change of basis). This means that there exists an invertible matrix $\mathbf{U}$, such that $\mathbf{A} = \mathbf{U}^{-1}\mathbf{B}\mathbf{U}$ and the corresponding transform is given by $[\mathbf{x}\ \mathbf{v}]^\top = \mathbf{U}[\mathbf{y} - \mathbf{y}_s\ \mathbf{a} - \mathbf{a}_s]^\top$. Assuming that $\mathbf{P}$ is invertible, we can write this equation as $\mathbf{UA} = \mathbf{BU}$. To solve this, we consider a block matrix representation of $\mathbf{U}$ and find the following solution,

$$\mathbf{U} = \begin{bmatrix}\mathbf{D}\left(\frac{\sqrt{\mathbf{a}_s}\odot\boldsymbol{\tau}_y}{\mathbf{y}_s}\right) & \mathbf{0}\\ -\mathbf{D}\left(\frac{\mathbf{a}_s}{\mathbf{y}_s}\right) & -\frac{1}{2}\mathbf{I}\end{bmatrix} \tag{122}$$

This change of basis gives us the transformation,

$$\begin{bmatrix}\mathbf{x}\\ \mathbf{v}\end{bmatrix} = \begin{bmatrix}\mathbf{D}\left(\frac{\sqrt{\mathbf{a}_s}\odot\boldsymbol{\tau}_y}{\mathbf{y}_s}\right) & \mathbf{0}\\ -\mathbf{D}\left(\frac{\mathbf{a}_s}{\mathbf{y}_s}\right) & -\frac{1}{2}\mathbf{I}\end{bmatrix}\begin{bmatrix}\mathbf{y} - \mathbf{y}_s\\ \mathbf{a} - \mathbf{a}_s\end{bmatrix} \tag{123}$$

or,

$$\begin{aligned}\mathbf{x} &= \frac{\sqrt{\mathbf{a}_s}\odot\boldsymbol{\tau}_y}{\mathbf{y}_s} \odot (\mathbf{y} - \mathbf{y}_s)\\ \mathbf{v} &= -\frac{\mathbf{a}_s}{\mathbf{y}_s} \odot (\mathbf{y} - \mathbf{y}_s) - \frac{(\mathbf{a} - \mathbf{a}_s)}{2}\end{aligned} \tag{124}$$

Substituting these expressions into $V(\mathbf{x},\mathbf{v}) = \mathbf{x}^\top(\mathbf{TK})\,\mathbf{x} + \mathbf{v}^\top\mathbf{T}\mathbf{v}$, and assuming the diagonal entries of the matrix $\mathbf{T}$ to be t_i and substituting the diagonal entries of $\mathbf{K}$, $k_i \to \sqrt{a_{si}}/(\tau_{y_i}\tau_{ai})$, we get the energy function in terms of $\mathbf{y}$ and $\mathbf{a}$,

$$V(\mathbf{y},\mathbf{a}) = \sum_{i=1}^{n} t_i \left[\frac{\tau_{y_i}}{\tau_{ai}}\frac{{a_{si}}^{3/2}}{{y_{si}}^2}(y_i - y_{si})^2 + \frac{a_{si}}{{y_{si}}^2}\left(\sqrt{a_{si}}\,(y_i - y_{si}) + \frac{y_{si}}{2\sqrt{a_{si}}}(a_i - a_{si})\right)^2\right] \tag{125}$$

We notice that Taylor expanding the term $\sqrt{a_i}y_i$ about $\sqrt{a_{si}}y_{si}$ and ignoring the second order terms, we get,

$$\sqrt{a_i}y_i \approx \sqrt{a_{si}}y_{si} + \sqrt{a_{si}}\,(y_i - y_{si}) + \frac{y_{si}}{2\sqrt{a_{si}}}(a_i - a_{si}) \tag{126}$$

Therefore the energy function, $V(\mathbf{y},\mathbf{a})$, is given by,

$$V(\mathbf{y},\mathbf{a}) = \sum_{i=1}^{n} t_i \frac{a_{si}}{{y_{si}}^2}\left[\frac{\tau_{y_i}}{\tau_{ai}}\sqrt{a_{si}}\,(y_i - y_{si})^2 + \left(\sqrt{a_i}y_i - \sqrt{a_{si}}y_{si}\right)^2\right] \tag{127}$$

Notice that $\sqrt{a_{si}}y_{si} = b_i z_i$. This gives us the following expression for the energy function,

$$V(\mathbf{y},\mathbf{a}) = \sum_{i=1}^{n} t_i \frac{a_{si}}{{y_{si}}^2}\left[\frac{\tau_{y_i}}{\tau_{ai}}\sqrt{a_{si}}\,(y_i - y_{si})^2 + \left(\sqrt{a_i}y_i - b_i z_i\right)^2\right] \tag{128}$$

Further, for an ORGaNICs circuit containing one y and one a neuron, after removing the proportionality constants, the energy function is given by,

$$V(y,a) = \frac{\tau_y}{\tau_a}\sqrt{a_s}\,(y - y_s)^2 + (\sqrt{a}y - bz)^2 \tag{129}$$

After plugging in the steady-state values, we get,

$$V(y,a) = \frac{\tau_y}{\tau_a}\sqrt{b_0^2\sigma^2 + wb^2z^2}\left(y - \frac{bz}{\sqrt{b_0^2\sigma^2 + wb^2z^2}}\right)^2 + (\sqrt{a}y - bz)^2 \tag{130}$$

For this system, it is easy to verify that this is a valid Lyapunov function and is minimized by the dynamics of the circuit. We demonstrate that it has the properties of a Lyapunov function. $V(y_s, a_s) = 0$, this can be seen from Eq. 129. We further need to show that, $\dot{V}(y,a) < 0\ \forall\ y \neq y_s\ \&\ a \neq a_s$. Using Eq. 118 & 130 $\dot{V}(y,a)$, we can write the total time derivative of the energy to be,

$$\begin{aligned}\frac{\mathrm{d}V(y,a)}{\mathrm{d}t} &= \frac{\partial V}{\partial y}\frac{\mathrm{d}y}{\mathrm{d}t} + \frac{\partial V}{\partial a}\frac{\mathrm{d}a}{\mathrm{d}t} \\ &= -\frac{(2ya_s - 3a_s y_s + a y_s)^2\left(\sqrt{a_s}\tau_a - w y_s^2\tau_y + \tau_y\right)}{2a_s\tau_a\tau_y}\end{aligned} \tag{131}$$

Since $a_s > 0$ and,

$$\left(\sqrt{a_s}\tau_a - w y_s^2\tau_y + \tau_y\right) = \left(\sqrt{a_s}\tau_a + \tau_y\left(1 - \frac{wb^2z^2}{b_0^2\sigma^2 + wb^2z^2}\right)\right) > 0, \tag{132}$$

for all the choices of parameters and $\forall\ y \neq y_s, a \neq a_s$, we have $\dot{V}(y,a) < 0$, therefore, it is a valid Lyapunov function and can be interpreted as the energy that decreases with time via the dynamics of ORGaNICs.

I Training details

The code (written in PyTorch [90]) to produce all the results can be found at https://github.com/shivangrawat/organics-ml. For both the static input and the sequential input, we train ORGaNICs on the MNIST handwritten digit dataset [70], and to the best of our knowledge, it does not pose any privacy concern and has been used widely by the ML community freely. The simulations were performed on an HPC cluster. All of the models were trained on a single A100 (80GB) GPU. We use Adam optimizer [91] with default parameters for minimizing the loss function.

I.1 Static MNIST input

We performed a random split of 57,000 training samples and 3,000 validation samples and picked the model with the largest validation accuracy for testing. To make a direct comparison to SSN [53], we use the same design as theirs. First, we train an autoencoder (Table 3) to reduce the dimensionality of MNIST images to 40 by using a mean-squared loss function. Then, we use this 40-dimensional vector as input to ORGaNICs and train it using the cross-entropy loss function. We additionally make the input gain $\mathbf{b}$ dependent on the input $\mathbf{x}$, $\mathbf{b} = f(\mathbf{W}_{bx}\mathbf{x})$, where f is sigmoid. A layer of ORGaNICs is given by Eq. 14,

$$\begin{aligned}\boldsymbol{\tau}_y \odot \dot{\mathbf{y}} &= -\mathbf{y} + f(\mathbf{W}_{bx}\mathbf{x}) \odot (\mathbf{W}_{zx}\mathbf{x}) + \left(\mathbf{1} - \sqrt{\lfloor \mathbf{a} \rfloor}\right) \odot \mathbf{W}_r \mathbf{y} \\ \boldsymbol{\tau}_a \odot \dot{\mathbf{a}} &= -\mathbf{a} + \mathbf{b}_0^2 \odot \boldsymbol{\sigma}^2 + \mathbf{W}\ \left(\mathbf{y}^2 \odot \lfloor \mathbf{a} \rfloor\right)\end{aligned} \tag{133}$$

The "output" of a layer is the steady-state firing rate of the neuron with the positive receptive field, i.e., $\mathbf{y}_s^+ = \lfloor \mathbf{y}_s \rfloor^2$. We parameterize $\mathbf{W}_r$ to have a maximum singular value of 1 and instead of simulating the system to find the fixed point, we use the iterative Algorithm 1 with a maximum number of steps = 10. More details about the parameters are given in Table 4, kaiming initialization is used from [92]. Additional hyperparameters are given in Table 6. We train ORGaNICs in a single-layer setting with the number of E neurons encoding the input $N_1 = 50, 80$. We also train two-layer ORGaNICs (Table 5) with $N_1 = 120$ and $N_2 = 60$ neurons in each layer. The model is trained using backpropagation and takes approximately 10 min to fully train.

Table 3: Autoencoder

Layer	Shape	Nonlinearity
Input → encoder (layer-1)	784 × 360	ReLU
encoder (layer-1) → encoder (layer-2)	360 × 120	ReLU
encoder (layer-2) → embedding	120 × 40	sigmoid
embedding → decoder (layer-1)	40 × 120	ReLU
decoder (layer-1) → decoder (layer-2)	120 × 360	ReLU
decoder (layer-2) → output	360 × 784	sigmoid

Table 4: ORGaNICs parametrization

Parameter	Shape	Learned	Initialization
$\mathbf{W}_{zx}$	$N \times M$	yes	kaiming uniform
$\mathbf{W}_{bx}$	$N \times M$	yes	kaiming uniform
$\mathbf{W}_r$	$N \times N$	yes	identity
$\mathbf{W}$	$N \times N$	yes	ones
$\mathbf{b}_0$	N	yes	random normal
$\boldsymbol{\sigma}$	N	no	ones

Table 5: ORGaNICs

Layer	Shape	Nonlinearity
Input → ORGaNICs (layer-1)	$40 \times N_1$	None
ORGaNICs (layer-1) → ORGaNICs (layer-2)	$N_1 \times N_2$	None
ORGaNICs (layer-2) → fully-connected	$N_2 \times 10$	None

I.2 Permuted and Unpermuted sequential MNIST

We performed a random split of 57,000 training samples and 3,000 validation samples and picked the model with the largest validation accuracy for testing. The unpermuted sequential MNIST task is defined as follows: for a given 28×28 image, we flatten it to get a one-dimensional, 784 timestep-long input. Then these pixels are presented as an input ($\mathbf{x}_i$, one pixel at each time-step i) to the discretized

Table 6: Hyperparameters

Hyperparameter	Static MNIST	Sequential MNIST
Batch size	256	256
Learning rate	0.001	0.01
Weight decay	10^{-5}	10^{-5}

ORGaNICs circuit given by the equation,

$$\begin{aligned}
\mathbf{y}_{i+1} &= \mathbf{y}_i + \frac{\Delta t}{\boldsymbol{\tau}_y} \odot \left(-\mathbf{y}_i + \mathbf{b}_i \odot (\mathbf{W}_{zx}\mathbf{x}_i) + \left(\mathbf{1} - \mathbf{a}_i^+\right) \odot \mathbf{W}_r \left(\sqrt{\mathbf{y}_i^+} \right) \right) \\
\mathbf{a}_{i+1} &= \mathbf{a}_i + \frac{\Delta t}{\boldsymbol{\tau}_a} \odot \left(-\mathbf{a}_i + \mathbf{b}_{0,i}^2 \odot \boldsymbol{\sigma}^2 + \mathbf{W} \left(\mathbf{y}_i^+ \odot \mathbf{a}_i^{+2} \right) \right) \\
\mathbf{b}_{i+1} &= \mathbf{b}_i + \frac{\Delta t}{\boldsymbol{\tau}_b} \odot \left(-\mathbf{b}_i + f(\mathbf{W}_{bx}\mathbf{x}_i + \mathbf{W}_{by}\mathbf{y}_i + \mathbf{W}_{ba}\mathbf{a}_i) \right) \\
\mathbf{b}_{0,i+1} &= \mathbf{b}_{0,i} + \frac{\Delta t}{\boldsymbol{\tau}_{b_0}} \odot \left(-\mathbf{b}_{0,i} + f(\mathbf{W}_{b_0x}\mathbf{x}_i + \mathbf{W}_{b_0y}\mathbf{y}_i + \mathbf{W}_{b_0a}\mathbf{a}_i) \right)
\end{aligned} \tag{134}$$

When we're done presenting the pixels we use $\mathbf{y}_{784}^+ = \lfloor \mathbf{y}_{784} \rfloor^2$ to make the predictions. To make this more challenging we also train ORGaNICs on permuted sMNIST where we first permute the pixels of all the images in some random order and the rest of the task is the same. Instead of parametrizing $\boldsymbol{\tau}$, we parametrize $\Delta t/\boldsymbol{\tau}_y = 0.05 * f(\mathbf{p}_y)$, $\Delta t/\boldsymbol{\tau}_a = 0.01 * f(\mathbf{p}_a)$ and $\Delta t/\boldsymbol{\tau}_b = 0.1 * f(\mathbf{p}_b)$ and $\Delta t/\boldsymbol{\tau}_{b_0} = 0.1 * f(\mathbf{p}_{b_0})$, so we can control the dimensionless relative time constants. In practice, we find it is better to make the $\mathbf{a}$ neurons sluggish compared to $\mathbf{y}$. This is based on the intuition given by the two-dimensional phase portrait for different relative time constants Fig. 3. All the parameters (including $\mathbf{W}_r$) are unconstrained for this task with initialization specified in Table 7. Since ORGaNICs is stable, we did not need to use gradient clipping for training, which is commonly used for LSTMs. Additional hyperparameters are given in Table 6. The model is trained using backpropagation through time (BPTT) and takes approximately 20 hours to fully train.

Table 7: ORGaNICs parametrization

Parameter	Shape	Learned	Initialization
$\mathbf{W}_{zx}$	$N \times 1$	yes	kaiming uniform
$\mathbf{W}_{bx}$	$N \times 1$	yes	kaiming uniform
$\mathbf{W}_{by}$	$N \times N$	yes	kaiming uniform
$\mathbf{W}_{ba}$	$N \times N$	yes	kaiming uniform
$\mathbf{W}_{b_0x}$	$N \times 1$	yes	kaiming uniform
$\mathbf{W}_{b_0y}$	$N \times N$	yes	kaiming uniform
$\mathbf{W}_{b_0a}$	$N \times N$	yes	kaiming uniform
$\mathbf{W}_r$	$N \times N$	yes	identity
$\mathbf{W}$	$N \times N$	yes	ones
$\boldsymbol{\sigma}$	N	no	ones

J Supplementary figures

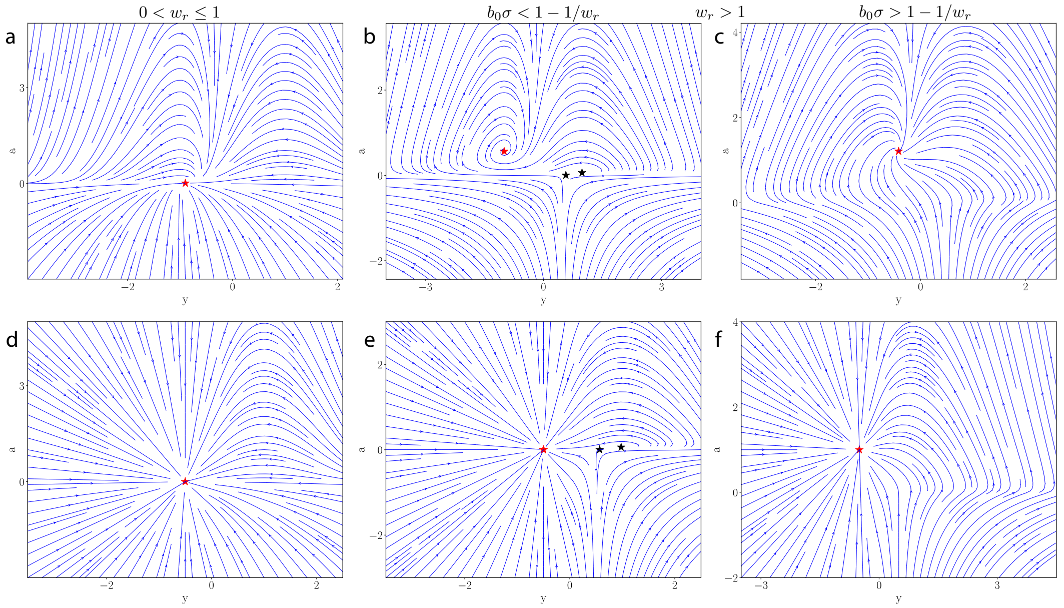

Figure 2: **Phase portraits for two-dimensional ORGaNICs under negative input drive. (a-c):** Results for the main model. **(d-f):** Results for the rectified model. Red stars and black stars indicate stable and unstable fixed points, respectively. The parameters for all plots are: $b = 0.5$, $\tau_a = 2\,\text{ms}$, $\tau_y = 2\,\text{ms}$, $w = 1.0$, and $z = -1.0$. For **(a) & (d)**, the parameters are $w_r = 0.5$, $b_0 = 0.5$, $\sigma = 0.1$; for **(b) & (e)**, $w_r = 2.0$, $b_0 = 0.5$, $\sigma = 0.1$; and for **(c) & (f)**, $w_r = 2.0$, $b_0 = 1.0$, $\sigma = 1.0$.

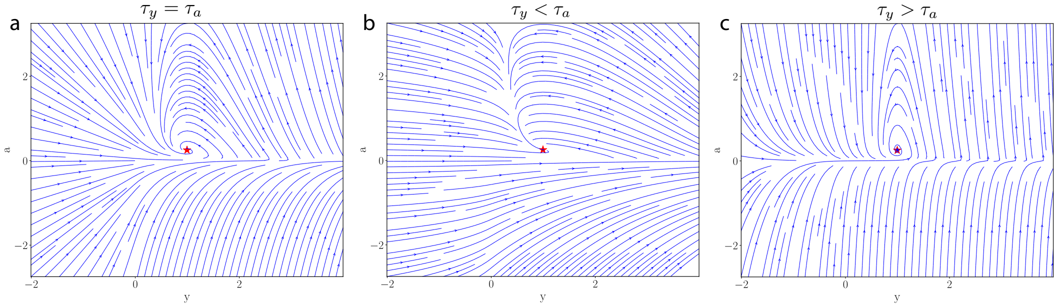

Figure 3: **Phase portraits for the rectified two-dimensional ORGaNICs for different time constants.** Red stars and black stars indicate stable and unstable fixed points, respectively. The parameters for all plots are: $w_r = 1.0$, $b_0 = 0.5$, $b = 0.5$, $\sigma = 0.1$, $w = 1.0$, and $z = 1.0$. For **(a)**, the time constants are $\tau_a = 2\,\text{ms}$, $\tau_y = 2\,\text{ms}$; for **(b)**, $\tau_a = 10\,\text{ms}$, $\tau_y = 2\,\text{ms}$; for **(c)**, $\tau_a = 2\,\text{ms}$, $\tau_y = 10\,\text{ms}$.

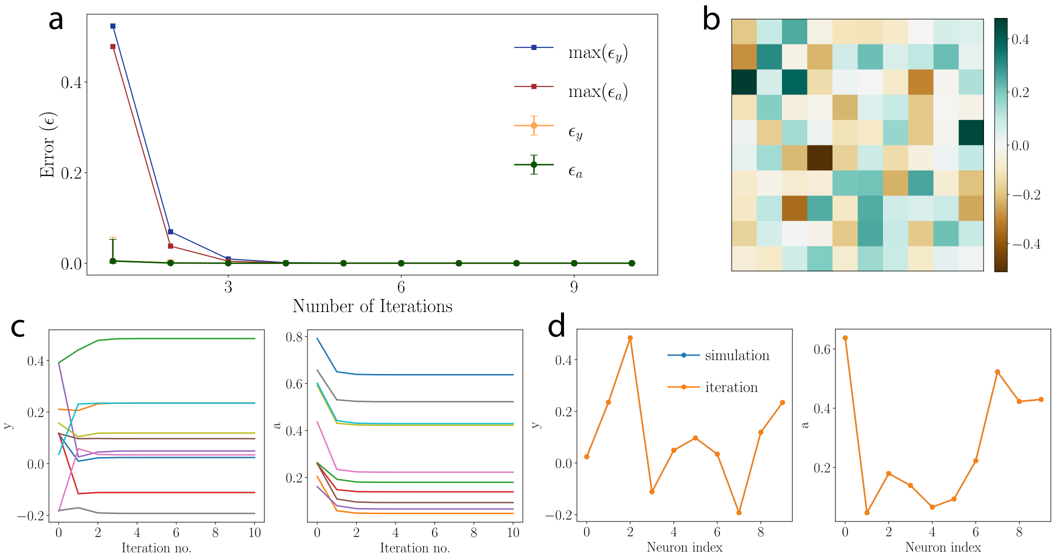

Figure 4: **Fast convergence of the iterative algorithm.** The results are for 20-dimensional ORGaNICs (10 E and 10 I neurons) with random parameters and inputs with the additional constraint of the maximum singular value of $\mathbf{W}_r$ equal to 1 and $||\mathbf{z}|| < 1$. **(a):** Mean (with error bars representing 1-sigma S.D.) and maximum errors (ϵ) as a function of number of iterations. ϵ is calculated as the norm of the difference between the true solution (found by simulation starting with random initialization) and the iteration solution. **(b):** An example of a randomly sampled $\mathbf{W}_r$. **(c):** Steady state approximation as a function of iteration number. Different lines represent different neurons. **(d):** Overlap between the iteration solution (after 10 iterations) and the true solution.

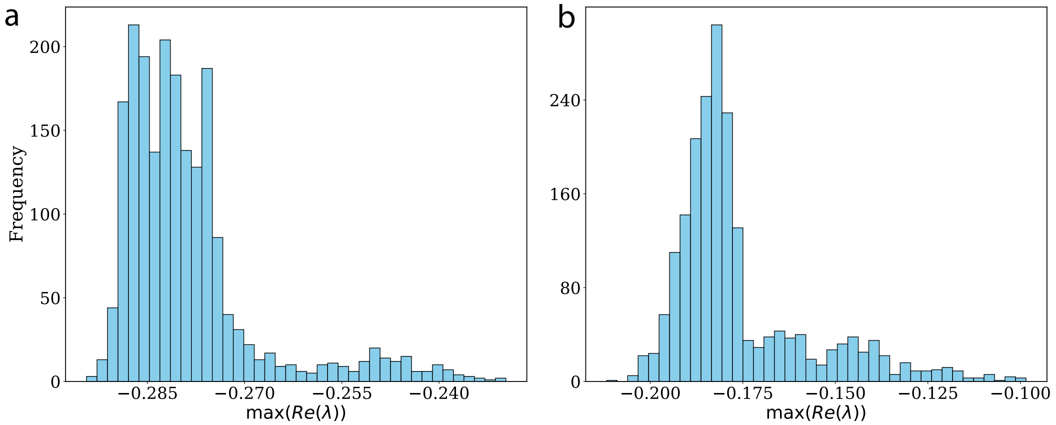

Figure 5: **Histogram for the eigenvalue with the largest real part**. We train two-layer ORGaNICs ($\tau_a = \tau_y = 2ms$) with a static MNIST input where $\mathbf{W}_r$ is constrained to have a maximum singular value of 1. We plot the histogram of eigenvalues of the Jacobian matrix with the largest real part, for different inputs from the test set. **(a):** Histogram for the first layer. **(b):** Histogram for the second layer. Note that since this is implemented in a feedforward manner (no feedback connections between layers), this is a cascading system with no feedback and hence we can perform the stability analysis of the two layers independently. this implies that all the eigenvalues of the Jacobian have negative real parts, implying asymptotic stability.

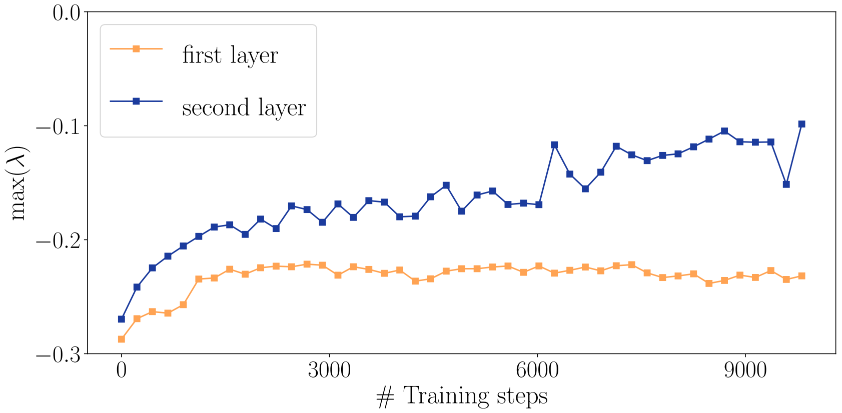

Figure 6: **Maximum real part of eigenvalue, across test samples, during training on static input (MNIST) classification task.** Since the maximum real part of the eigenvalue remains less than 0 throughout training, it indicates that the system remains stable.

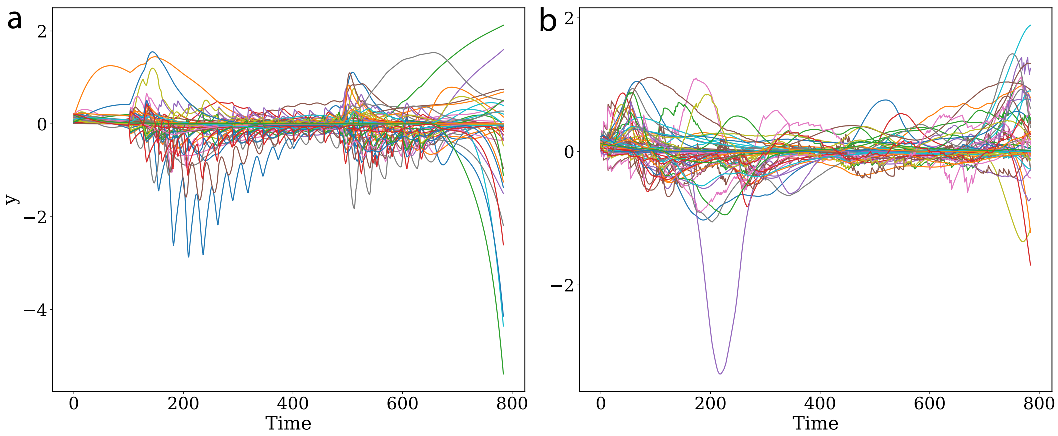

Figure 7: **Trajectories of $\mathbf{y}$ for a random input from the test set when ORGaNICs is trained as an RNN**. **(a):** Unpermuted sequential MNIST, **(b):** Permuted sequential MNIST.
